# Quantitative Assessment of Labeling Probes for Super‐Resolution Microscopy Using Designer DNA Nanostructures

**DOI:** 10.1002/cphc.202100185

**Published:** 2021-05-04

**Authors:** Mahipal Ganji, Thomas Schlichthaerle, Alexandra S. Eklund, Sebastian Strauss, Ralf Jungmann

**Affiliations:** ^1^ Faculty of Physics and Center for Nanoscience, LMU Munich Geschwister-Scholl-Platz 1 80539 Munich Germany; ^2^ Max Planck Institute of Biochemistry Am Klopferspitz 18 82152 Martinsried Germany; ^3^ Current Address: Department of Biochemistry Indian Institute of Science CV Raman Road 560012 Bengaluru India; ^4^ Current Address: Department of Biochemistry Institute for Protein Design University of Washington Seattle WA USA

**Keywords:** DNA origami, DNA-PAINT, labeling probes, single-molecule imaging, super-resolution microscopy

## Abstract

Improving labeling probes for state‐of‐the‐art super‐resolution microscopy is becoming of major importance. However, there is currently a lack of tools to quantitatively evaluate probe performance regarding efficiency, precision, and achievable resolution in an unbiased yet modular fashion. Herein, we introduce designer DNA origami structures combined with DNA‐PAINT to overcome this issue and evaluate labeling efficiency, precision, and quantification using antibodies and nanobodies as exemplary labeling probes. Whereas current assessment of binders is mostly qualitative, e. g. based on an expected staining pattern, we herein present a quantitative analysis platform of the antigen labeling efficiency and achievable resolution, allowing researchers to choose the best performing binder. The platform can furthermore be readily adapted for discovery and precise quantification of a large variety of additional labeling probes.

## Graphical abstract

**Probe performance**: Using programmable, antigen‐decorated designer DNA origami nanostructures, performance of labeling probes, including antibodies and nanobodies, is tested. The platform reveals that primary‐secondary antibody labeling can only resolve antigens spaced down to 40 nm. The nanostructures enable performance evaluation of a multitude of labeling probes for super‐resolution microscopy.

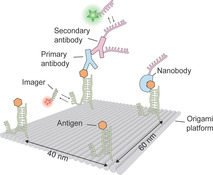

Super‐resolution microscopy has revolutionized research in the life sciences by circumventing the diffraction limit of light.^[1]^ Current state‐of‐the‐art implementations technically achieve molecular‐scale resolution (better than 5 nm)^[2]^ and enable quantitative imaging.^[3]^ However, translating these capabilities to cellular protein imaging has been hindered by the lack of small, efficient, and ubiquitously available labeling probes. To overcome this, novel approaches involving nanobodies,^[4]^ genetically encoded self‐labeling tags (e. g. SNAP and Halo),^[5]^ small protein scaffolds (e. g. affimers or iris probes),^[6]^ or aptamers^[7]^ were implemented. While developing suitable binders for super‐resolution applications has become of paramount importance,^[8]^ it is currently difficult to quantitatively assess e. g. their labeling efficiency and achievable spatial resolution in a straightforward, modular, and sample‐unbiased way. To partly address this, cell lines featuring genetically‐encoded tags fused to Nuclear Pore Complex (NPC) proteins were developed.^[9]^ However, these gene‐edited cells are time‐consuming to construct and currently only cover genetically‐encodable tags as potential labeling probes. Furthermore, biological heterogeneity in NPC structure and assembly state might lead to additional evaluation uncertainty. A previous study employed DNA origami nanostructures to quantify protein copy numbers in STORM super‐resolution microscopy by analyzing the binding and blinking behavior of AlexaFluor647‐labeled secondary antibodies to primary antibodies binding to GFP molecules anchored on DNA origami.^[10]^ However, this approach is missing a ground truth measure of the super‐resolved antigen position. Additionally, quantification via counting of localizations in dye‐switching‐based SMLM can lead to over‐ and undercounting artefacts.^[11]^ In this regard, previous work on labeling probe evaluation for super‐resolution microscopy has so far neglected the influence of the probe on the achievable distinct separation of single antigen positions.

To address these issues, we here introduce a DNA‐PAINT‐based single‐molecule assay featuring designer DNA origami structures as platforms for quantitative assessment of labeling probes. Our approach allows us to correlate the true position of the antigen with the binder and thus enables absolute quantification of labeling efficiency, stoichiometry, probe‐size‐dependent achievable spatial resolution, and further aspects such as multivalency.^[12]^ Based on the specific antigens and binders tested in this study, we find that antibody‐based labeling results in poor efficiency and prevents the dissection of nanoclusters with antigens spaced closer than 40 nm, approximately 10‐times larger than achievable spatial resolution with current state‐of‐the‐art super‐resolution approaches.^[2]^ We note that we do not generally suggest that antibodies are inferior binders, however we want to emphasize the usability of our approach to quantitatively assess the binder performance in order to select the most ideal probe for a specific target antigen and application.

We first developed a single‐molecule assay to evaluate labeling efficiency, localization precision, and stoichiometry of different probes (Figure 1). Our assay employs surface‐bound, DNA‐conjugated antigens (Figure 1a), where one section of the DNA oligonucleotide is used for stable hybridization to a surface‐immobilized strand. A second sequence extension enables DNA‐PAINT imaging, probing the antigen's presence and localizing its true position (green single‐stranded extension in Figure 1a). After immobilization, antigens are targeted using DNA‐conjugated binders such as antibodies or nanobodies, carrying orthogonal DNA‐PAINT docking sequences (depicted in magenta in Figure 1a). DNA‐PAINT imaging is carried out using two spectrally distinct dyes (ATTO647 N for antigen position and Cy3B for binder localization), enabling the direct quantification of efficiency, precision, and stoichiometry. We first assayed the performance of polyclonal antibodies targeting digoxigenin (DIG) and an eight‐amino acid 5‐phosphorylated C‐terminal domain (1×CTD) of the RNA polymerase. The antibody for the CTD domain was selected via an initial DNA‐PAINT imaging experiment of RNA polymerase in HeLa cells, which showed specific staining in the nucleus (Figure S1 in the Supporting Information). We also tested 5× repeats of the CTD antigen (5×CTD) to evaluate potential effects of multivalency. We furthermore probed the performance of the ALFA‐tag and its corresponding nanobody.^[13]^ After acquisition of the antigen and binder position using orthogonal ATTO647N‐ and Cy3B‐labeled imager strands, we aligned the two channels (Figure 1b and Figures S2–12) and calculated the labeling efficiency using a co‐localization analysis of both antigen and probe signals (Figure 1c, see methods for analysis details). Surprisingly, we observed relatively low labeling efficiencies of 25 % and 8 % for DIG and 1×CTD. We observed a moderate improvement in the labeling efficiency, when we replaced the secondary antibodies with secondary nanobodies in the case of 1×CTD, indicating that the poor labeling efficiency is mainly due to limitations of the primary antibody. Efficiency increased to 63 % in the case of 5×CTD labeled with primary and secondary antibodies, suggesting most likely multivalent binding of two antigens by a single primary antibody.^[12]^ In contrast, the efficiency for the ALFA‐tag‐nanobody system was 77 %. To put these numbers into context, we compared the results to a “perfect” labeling scenario by replacing the antigen with a direct single‐stranded DNA extension (Figure 1a, rightmost cartoon). In this case, we observed 82 % co‐localization, in good agreement with earlier studies.^[14]^ Assuming this labeling efficiency as upper bound, we rescaled the apparent labeling efficiency for the antibodies and nanobody to 30, 10, 16, 78, and 94 %, for DIG, 1×CTD, 1×CTD labeled with primary antibody and secondary nanobody, 5×CTD, and ALFA‐tag respectively, highlighting the close‐to‐perfect labeling efficiency of the nanobody system. Next, we evaluated the effect of the labeling probe on the size of the localization cloud in a corresponding localization microscopy image of individual, well‐separated antigens (Figure 1d). While the ALFA‐tag and direct extension yielded a similar precision (3.5 nm and 3.9 nm), the primary and secondary antibody‐labeled 1×CTD and 5×CTD performed considerably worse with precisions of 8.2 nm and 10.1 nm, as expected from the size of the antibody sandwiches and the multiple DNA docking sites attached to them. We then assessed probe performance for quantitative imaging by evaluating the total number of docking strands per antigen. For this, we used qPAINT,^[3]^ which allowed us to directly count integer numbers of strands per target. In addition to the DNA‐conjugated antigens, we surface‐immobilized DNA origami structures carrying twelve docking strands for qPAINT calibration. By comparative analysis of binding kinetics on antigens and DNA origami structures (Figure S13**)**, we obtained the absolute number of strands associated with the antigens. We first quantified this for the 1×CTD antigen (Figure 1e), labeled with primary and DNA‐conjugated secondary antibodies using a 2 : 1, 5 : 1, and 10 : 1 excess ratio of DNA over secondary antibody. We observed an average of 6, 10, and 19 binding sites (Figure 1e), which (if not initially calibrated) would lead to overcounting of antigens per single target. Next, we evaluated the stoichiometry in the case of the ALFA‐tag‐nanobody system, where we would expect a maximum of two binding sites per ALFA‐tag, as the corresponding nanobody carries two cysteine residues available for DNA conjugation (Figure 1f). Indeed, we observed either one or two sites per ALFA‐tag, improving suitability for quantitative imaging.

**Figure 1:**
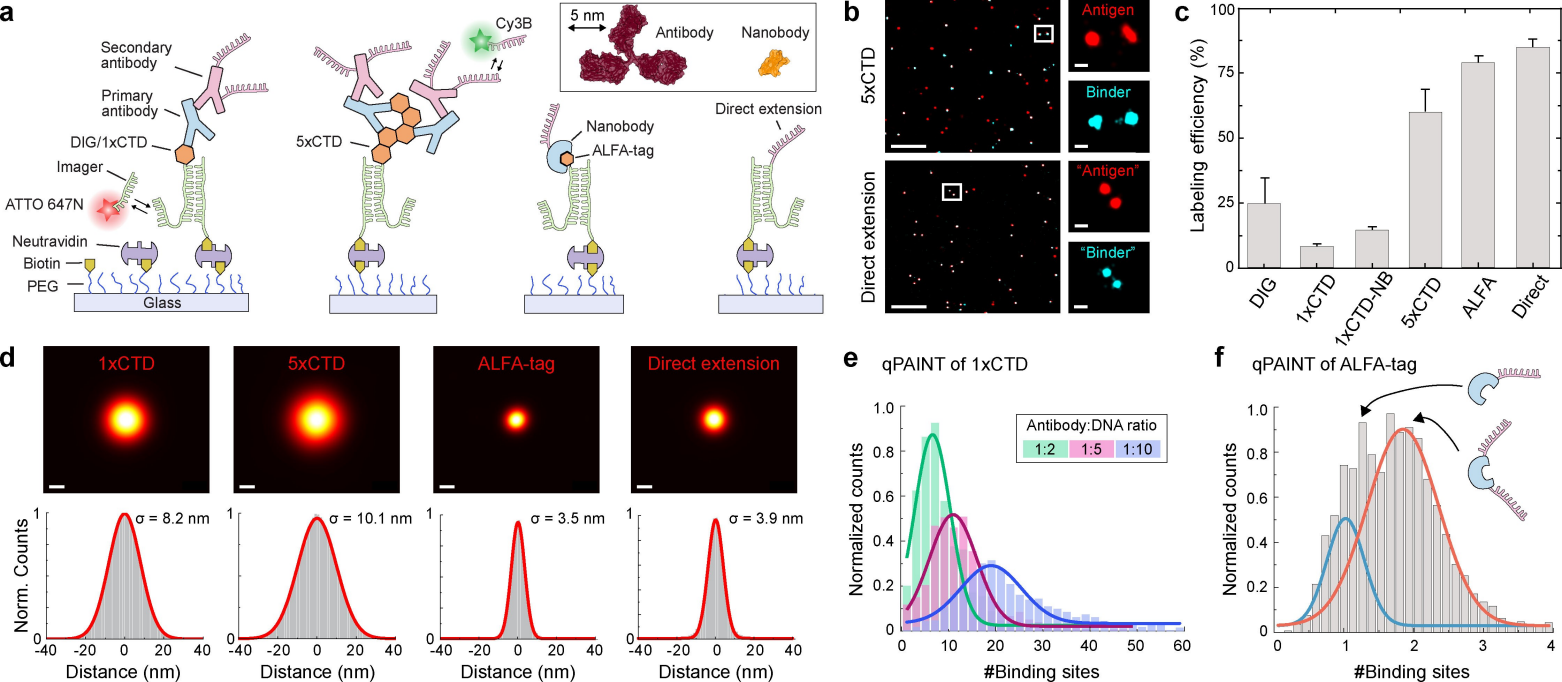
Single‐molecule assay for quantifying labeling probe performance. a) Surface‐immobilized DNA‐conjugated antigens (DIG, 1×CTD, 5×CTD, and ALFA‐tag) are targeted using DNA‐conjugated nanobodies or primary and DNA‐conjugated secondary antibodies or nanobodies. Antigens and corresponding probe positions are independently visualized using orthogonal DNA‐PAINT imager strands. b) Representative co‐localization data of antigen and labeling probe (top: 5×CTD, bottom: direct extension). Zoom‐in of selected areas show co‐localization between the antigen and binder. c) Labeling efficiency for different probes from (a) (n≥500 for each probe, three independent experiments. Here 1×CTD‐NB refers to labeling with primary antibody and secondary nanobody. Error bars represent standard deviation of three independent measurements). d) Center‐of‐mass‐aligned localizations of single probes (top row) and corresponding cross‐sectional histograms (bottom) for different antigen‐binder system (n≥500 for each probe, solid lines are Gaussian fits). e) qPAINT analysis to quantify the number of docking strands for individual 1×CTD antigen labeled with DNA‐conjugated secondary antibodies. Antibody conjugation was performed with 1 : 2 (green), 1 : 5 (red), and 1 : 10 (blue) ratio of antibody to docking strand (n≥500 for each ratio, solid lines are Gaussian fits). f) qPAINT quantification of DNA‐conjugated ALFA‐tag nanobodies shows two distinct populations (corresponding to one and two binding sites, solid lines are a two‐component Gaussian fit). Scale bars: 500 nm (panel (b) overview), 40 nm (panel (b), zoom‐ins), and 10 nm (d).

The single‐molecule immobilization assay enabled us to quantitatively assess the achievable labeling efficiency, stoichiometry, and localization precision. However, while quantitative evaluation and subsequent optimization of these parameters are important prerequisites, they do not directly translate to achievable spatial resolution in “real‐world” settings where antigens are not sparsely distributed. To evaluate denser, more controlled nanoclusters, we designed DNA origami structures^[15]^ (Figure S14 and Table S1**)** carrying antigens arranged in different spatial nanopatterns (Figure 2). We used three different antigen binding patterns on DNA origami (Figure 2a): A 60×40 nm four‐point pattern, 30‐nm, and 20‐nm‐grids. The positions of DNA‐conjugated antigens were visualized in addition to the locations of their respective binders using Exchange‐PAINT^[16]^ (Figures S15–S32). In order to assess the resolution capabilities of each antigen‐binder pair in an unbiased way, we aligned and summed up localizations of approximately 100 structures and compared the achievable spatial resolutions in a cross‐sectional histogram analysis between the antigen positions (Figure 2b) and their corresponding binder positions (Figure 2c and 2d). Interestingly, while the 60‐nm‐spaced 1×CTD antigens were well‐resolved by primary and secondary antibodies (Figure 2c, top), the 40‐nm spacing approached the resolution limit, and the 30‐ and 20‐nm spaced patterns (Figure 2c, center and bottom) were unresolvable. The ALFA‐tag‐nanobody system, however, was able to resolve all patterns (Figure 2d) and retained the high‐labeling efficiency to distinguish single sites in a nanocluster (Figure S33). Replacing secondary antibodies with secondary nanobodies, the 40‐nm spacing was resolved, but the 20‐nm and 30‐nm spaced patterns still remained unresolvable, despite the smaller secondary nanobody size compared to the antibody (Figures S27–S32, S34). The antibody‐induced broadening of a single antigen location from single‐molecule data – a commonly used proxy for spatial resolution – suggests that 20 nm resolution (see Figure 2d) should be achievable. However, our DNA origami platform reveals that this distance cannot be resolved, possibly due to the heterogenous nature of the (polyclonal) antibody localization clusters arising from single sites.

**Figure 2:**
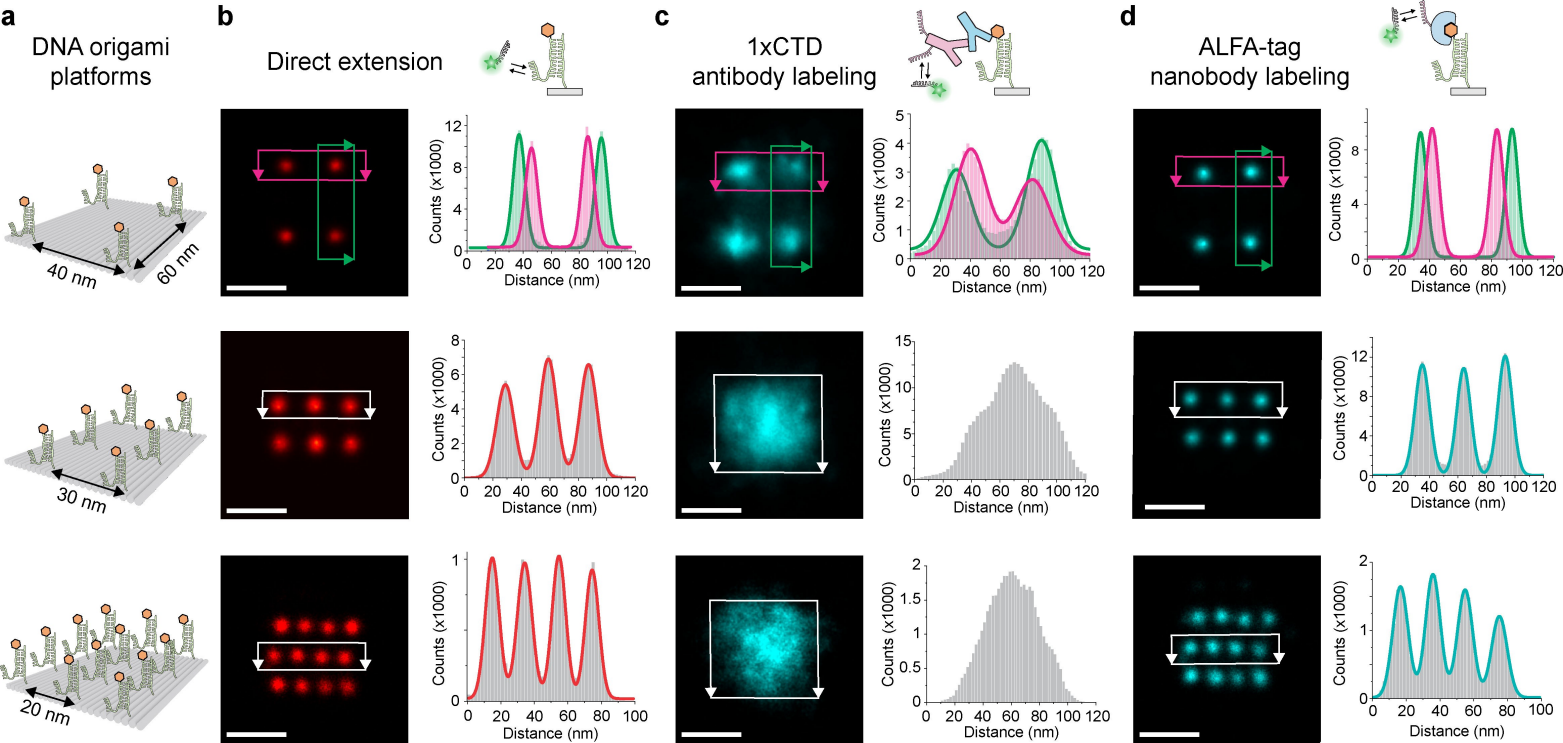
Designer DNA origami platform for assessment of binder‐dependent spatial resolution. a) Schemes of DNA origami structures with positions of antigens (orange). b–d) Top row: Schemes for DNA‐PAINT imaging of antigen position (b), antibody positions bound to 1×CTD (c), and ALFA‐tag nanobody positions (d). DNA origami sum images of antigen positions (red, b), antibody positions (cyan, c), ALFA‐tag nanobody positions (cyan, d) and corresponding cross‐sectional histogram analysis. Solid lines are multi‐component Gaussian fits to data from the highlighted rectangular regions with the corresponding color scheme of the origami sum images on the left side to them. (n>100 for each structure). Scale bars: 50 nm.

We here introduce a DNA‐PAINT‐based single‐molecule assay featuring designer DNA origami nanostructures as programmable platforms to quantitatively assess the performance of labeling probes for super‐resolution microscopy. Using this approach, we showed that antibody‐based labeling can be prone to artifacts, including poor labeling efficiency, non‐stoichiometric labeling, and increased linkage errors, lowering the achievable spatial resolution. Labeling of antigens using primary and secondary antibodies results in a large localization cluster (>30 nm), masking underlying nanopatterns. Furthermore, the combination of non‐stoichiometric labeling and low efficiency of antibody probes only enable limited quantification. In conclusion, resolving antigens spaced closer than 30 nm combined with accurate quantification requires small and stoichiometric labeling probes such as nanobodies. Our results further suggest that a careful evaluation of each labeling system is mandatory for best‐performing super‐resolution microscopy. Our designer DNA origami labeling platform enables unbiased binder characterization and – for the first time – a quantitative measure for binder‐dependent localization precision and accuracy. In the future, the platform could be employed in highly multiplexed, barcoded binder discovery assays,^[17]^ involving a large variety of platform‐bound antigens. Further applications could involve qPAINT and single‐antigen‐resolution‐imaging on DNA origami to precisely discover and quantify multivalent binders for biomedical applications.

## Conflict of interest

R. J. is cofounder of Ultivue Inc. and R. J. and S. S. are cofounders of Massive Photonics GmbH.

## Supporting information

As a service to our authors and readers, this journal provides supporting information supplied by the authors. Such materials are peer reviewed and may be re‐organized for online delivery, but are not copy‐edited or typeset. Technical support issues arising from supporting information (other than missing files) should be addressed to the authors.

Supplementary
