# Supplementary material for: Quantitative Assessment of Labeling Probes for Super‐Resolution Microscopy Using Designer DNA Nanostructures
Source: Chemphyschem. 2021 May 4;22(10):911–4. doi: 10.1002/cphc.202100185 (PMC8251534; doi:10.1002/cphc.202100185)
Supplement: Supplementary file 1 — Supplementary [file CPHC-22-911-s001.pdf]

ChemPhysChem

Supporting Information

Quantitative Assessment of Labeling Probes for Super-Resolution Microscopy Using Designer DNA Nanostructures

Mahipal Ganji+, Thomas Schlichthaerle+, Alexandra S. Eklund, Sebastian Strauss, and Ralf Jungmann*

Supplementary Information

Table of Contents

Materials and Methods	
Supplementary Figure 1	DNA-PAINT imaging of the CTD domain of RNA polymerase using primary and DNA-conjugated secondary nanobodies in HeLa cells
Supplementary Figure 2	Overview image of DIG single-molecule experiments
Supplementary Figure 3	Single DIG sites selected for sum image from single-molecule experiments
Supplementary Figure 4	Overview image of 1xCTD single-molecule experiments
Supplementary Figure 5	Single 1xCTD sites selected for sum image and probe-based resolution estimate from single-molecule experiments
Supplementary Figure 6	Overview image of 5xCTD repeat single-molecule experiments
Supplementary Figure 7	Single 5xCTD repeat sites selected for sum image and probe-based resolution estimate from single-molecule experiments
Supplementary Figure 8	Overview image of 1xCTD repeat single-molecule experiments imaged with primary antibody and secondary nanobody
Supplementary Figure 9	Overview image of ALFA-tag based single-molecule experiments
Supplementary Figure 10	Single ALFA-tag sites selected for sum image and probe-based resolution estimate from single-molecule experiments
Supplementary Figure 11	Overview image of directly extended DNA-PAINT docking strand single molecule experiments
Supplementary Figure 12	Single directly extended sites selected for sum image and probe-based resolution estimate from single molecule experiments
Supplementary Figure 13	qPAINT comparison of a single ALFA-tag site with 20-nm-grid DNA origami calibration standard
Supplementary Figure 14	Overview of DNA origami structures
Supplementary Figure 15	Overview image of 1xCTD nanoclusters templated at 40 and 60 nm distances
Supplementary Figure 16	Selected 1x CTD nanoclusters templated at 40x60nm distances for probe-based resolution estimate and labeling shadow determination
Supplementary Figure 17	Overview image of 1xCTD nanoclusters templated at 30 nm distances
Supplementary Figure 18	Selected 1x CTD nanoclusters templated at 30 nm distances for probe-based resolution estimate and labeling shadow determination
Supplementary Figure 19	Overview image of 1xCTD nanoclusters templated at 20 nm distances
Supplementary Figure 20	Selected 1x CTD nanoclusters templated at 20 nm distances for probe-based resolution estimate and labeling shadow determination
Supplementary Figure 21	Overview image of ALFA-tag nanoclusters templated at 40 and 60 nm distances.
Supplementary Figure 22	Selected ALFA-tag nanoclusters templated at 40 and 60 nm distances for probe-based resolution estimate and labeling shadow determination
Supplementary Figure 23	Overview image of ALFA-tag nanoclusters templated at 30 nm distances

**Supplementary Figure 24**	**Selected ALFA-tag nanoclusters templated at 30 nm distances for probe-based resolution estimate and labeling shadow determination**
**Supplementary Figure 25**	**Overview image of ALFA-tag nanoclusters templated at 20 nm distances**
**Supplementary Figure 26**	**Selected ALFA-tag nanoclusters templated at 20 nm distances for probe-based resolution estimate and labeling shadow determination**
**Supplementary Figure 27**	**Overview image of 1xCTD nanoclusters templated at 40x60 nm distances labeled with primary antibody and secondary nanobody**
**Supplementary Figure 28**	**Selected 1xCTD nanoclusters templated at 40x60 nm distances for probe-based resolution estimate and labeling shadow determination**
**Supplementary Figure 29**	**Overview image of 1xCTD nanoclusters templated at 30 nm distances labeled with primary antibody and secondary nanobody**
**Supplementary Figure 30**	**Selected 1x CTD nanoclusters templated at 30 nm distances for probe-based resolution estimate and labeling shadow determination**
**Supplementary Figure 31**	**Overview image of 1xCTD nanoclusters templated at 20 nm distances labeled with primary antibody and secondary nanobody**
**Supplementary Figure 32**	**Selected 1x CTD nanoclusters templated at 20 nm distances and labeled with primary antibody and secondary nanobody for probe-based resolution estimate and labeling shadow determination**
**Supplementary Figure 33**	**Evaluation of Alfa-tag labeling efficiency on templated nanoclusters**
**Supplementary Figure 34**	**DNA origami assessment of primary antibodies and secondary nanobodies**
**Supplementary Table 1**	**DNA strands for DNA origami folding**
**Supplementary Table 2**	**Additional DNA reagents for imaging and peptide attachment**
**Supplementary Table 3**	**Peptide reagents**
**Supplementary Table 4**	**Imaging conditions**

Materials and Methods

Antibody labeling. Antibody labeling was performed as previously described[3] . In brief, 300 µl of 1 mg/ml secondary anti-rabbit antibody (Jackson Labs) was concentrated via 100 kDa molecular weight cutoff (MWCO) centrifugal filter unit (Merck Millipore, cat: UFC510024) and the volume was adjusted to 100 µl in 1×PBS, pH 7.2. A 10× molar excess of Dibenzocyclooctyne-*N*-hydroxysuccinimidyl ester (DBCO-NHS) crosslinker in 5 µl DMF was added to 100 µl of concentrated secondary antibodies and reacted for 2 hours at 4 °C on a shaker in the dark. Afterwards excess crosslinker was removed via 7 kDa MWCO Zeba desalting spin column (Thermo Fisher Scientific, cat: 89882). The antibody-crosslinker was then reacted with 10× molar excess of Azide-DNA (Metabion Inc.) for overnight at 4 °C on a shaker in the dark. Excess unreacted DNA was removed with 100 kDa MWCO centrifugal filter. The final volume was adjusted to 100 µl in 1×PBS and stored at 4 °C until usage. Final usage concentration was approximately 100 µg/ml.

Nanobody labeling. ALFA-Tag (clone:1G5) and anti-rabbit IgG (clone: 10E10) nanobodies (NanoTag Biotechnologies. Göttingen) with two cysteines one at C-terminus and other at N-terminus were conjugated with DNA as previously reported[19]. Nanobodies were reduced with 5 mM TCEP in 1× PBS containing 3 mM EDTA at pH 7.2 for 30 minutes in the dark at room temperature. Subsequently, reduced nanobody was buffer-exchanged into 1× PBS by using 10 kDa MWCO centrifugal filter and the volume was adjusted to 100 µl. A 20x molar excess DBCO-PEG4-Maleimide crosslinker (Jena Biosciences, cat: CLK-A108P-100), dissolved in DMF, was added to the ALFA-Tag nanobody and incubated for 2 h at 4 °C on a shaker. Unreacted DBCO crosslinker was removed via 10 kDa MWCO centrifugal filter. A 10x molar excess of Azide-DNA was incubated with the ALFA-Tag nanobody for overnight at 4 °C. Purification of free DNA and unlabeled ALFA-Tag nanobody was performed on a ÄKTA pure system (Cytiva) using 1 ml RESOURCE Q (Cytiva, cat: 17-1177-01) column. Appropriate fractions were concentrated and buffer-exchanged into PBS via 10 kDa MWCO centrifugal filter.

Preparation of PEG-passivated surfaces. Poly-ethelyne-glycol (PEG) passivated microscope coverslips were prepared as described earlier[24]. Briefly, the coverslips and glass slides were sonicated in acetone, water, and 1M KOH successively for around 20 minutes each. Both the coverslips and glass slides were then treated with Piranha (3:1 ratio of H_2SO_4 and H_2O_2) for 30 minutes. After this, they were washed with MilliQ and sonicated in methanol for 20 minutes before placing them in aminosilanization solution composed of 20:1:2 of methanol:acetic acid: 3-aminopropyl trimethoxysilane (Roth, cat: 2328.1). Both the coverslips and slides were washed twice with methanol, followed by MilliQ water, and dried with dry N_2 gas. 75 mg of NHS-ester mPEG (5,000 Da, LaysonBio, cat: Biotin-MPEG-SVA-5000-1g and 25 mg of biotinylated NHS-PEG (5,000 Da, LaysonBio, cat: MPEG-SVA-5000-1g) was dissolved in a freshly prepared 0.1 M Sodium Bicarbonate buffer with pH 8.5. 80 µl of this solution was placed on a glass slide that is lying flat, over which a coverslip was placed, and the sandwich was incubated overnight in dark and humid conditions. On the next day, the coverslips and slides were washed with MilliQ water and dried with dry N_2 gas. The coverslips and slides were then reacted with Methyl- $(PEG)_4$ - NHS-ester (333 Da, Invitrogen, cat: 22341) in 0.1 M Sodium Bicarbonate buffer for around 1 hour. After rinsing with MilliQ and drying with N_2 gas, coverslips and slides were stored at -80 °C until usage (typically less than 3 months).

Peptide preparation. Three peptides, 1xCTD, 5xCTD, and ALFA-Tag, were synthesized at the peptide facility of MPI of Biochemistry (See **Supplementary Table** 3 for the peptide sequences). Peptides carried a C-terminal lysine-azide functional moiety for conjugation with DNA strands. A 20× molar excess of DBCO-DNA was reacted with the azide-modified peptide for 24 h at 4 °C in 1×PBS. Peptide-DNA conjugates were purified with a ÄKTA pure system using 1 ml RESOURCE Q column by applying a 30 min gradient of 1×PBS to 1 M NaCl. Peak fractions were afterwards concentrated and buffer-exchanged via 3 kDa MWCO spin filters. Resulting peptide-DNA conjugates were stored at -20 °C until usage.

DNA origami preparation. For folding DNA origami, 10 nM M13 single-stranded DNA scaffold, 100 nM of each staple, 150 nM of staples with S1 extension, 1500 nM of peptide-conjugated S1-handle with an extension carrying P3 docking site, and 1000 nM of biotin-conjugated staples were pooled (see **Supplementary Table 1**) into 50 µl of 10 mM Tris-HCl, pH 8.0, 0.2 mM EDTA, 150 mM NaCl, 10 mM MgCl2 buffer. Structures were thermally annealed in a Thermocycler (Eppendorf Inc.) by gradual cooling the mixture at a rate of 1 °C per 3 minutes from 60 °C to 4 °C. The folded origami structures were then purified from excess staples using 100 kDa MWCO centrifugal filters. Purified origami structures were stored in buffer C (PBS, 500 mM NaCl) at -20 °C until usage. A similar scheme was used for folding reference origami for qPAINT analysis with following pool of staples: 10 nM M13 single-stranded DNA scaffold, 100 nM of each staple, 200 nM of staples with P1 docking site extension, and 1000 nM of biotin-conjugated staples were pooled into 50 ul of 10 mM Tris-HCl, pH 8.0, 0.2 mM EDTA, 150 mM NaCl, 10 mM $MgCl_2$ buffer.

Microscopy Setup. Fluorescence imaging was carried out on an inverted microscope (Nikon Instruments, Eclipse Ti and Ti2) with the Perfect Focus System, applying an objective-type TIRF configuration with an oil-immersion objective (Apo SR TIRF 100×, NA 1.49, Oil). TIRF/Hilo angle was adjusted for optimum signal-to-noise ratio when imaging. 561-nm and 642-nm lasers (Coherent, Sapphire, Toptica iBeam smart, and MPB DPSS) were used for excitation. The laser beams were passed through cleanup filters (Chroma Technology, ZET561/10 and ZET 640/10) and coupled into the microscope objective using a beam splitter (Chroma Technology, ZT561rdc and ZT640rdc). Fluorescence was spectrally filtered with an emission filter (Chroma Technology, ET600/50m and ET700/75m) and imaged

on an sCMOS camera (Andor, Zyla 4.2 Plus) without further magnification, resulting in an effective pixel size of 130 nm (after 2 × 2 binning).

DNA-PAINT imaging. Flow chambers were assembled by sandwiching double-sided tape in between PEG-passivated glass slide and cover slip with a volume of around 10 µL. Flow chambers contained 1 mm holes at each end for buffer exchange. A tubing with syringe was inserted at one end and sealed with 5-minute epoxy. A 20-200 µL pipette tip was inserted at the other end for introducing buffers. First, a 0.1 mg/ml of neutravidin (Invitrogen Inc.,) was introduced into the biotin-PEG/PEG passivated flow cell and incubated for 1 minute. Excess neutravidin was washed off with PBS.

In the case of single-molecule imaging, a pre-hybridized 250 pM biotin-S1/S1-antigen (1x CTD, 5xCTD, Digoxigenin, or ALFA-tag, see **Fig. 1a**) in PBS was incubated in the flow cell for 1 minute and excess product was removed with PBS. In the case of ALFA-tag, we incubated a 100 nM P1-conjugated ALFA-tag nanobody in 0.5x buffer C and 2 (w/v) % BSA for 30 minutes before washing off the unbound nanobody with buffer C.

For labeling of either the CTD or Digoxigenin, we incubated 60 nM rabbit primary antibody (abcam, cat: ab5131) against the respective antigen in 0.5x buffer C and 2 (w/v) % BSA for 60 minutes before washing off the excess antibody. We then incubated a 6 nM P1-conjugated anti-rabbit secondary antibody in 0.5x buffer C and 2 (w/v) % BSA for around 30 minutes. For the labeling with secondary nanobody, 60 nM of the same primary antibody was preincubated with 6 nM P1-conjugated anti-rabbit secondary nanobody for 5 minutes in 0.5x buffer C and 2(w/v) % BSA. Both probes were then introduced and incubated for 45 minutes. The sample was then washed with buffer C. Gold nanoparticles (Cytodiagnostics, cat: G-90-100) diluted 1:10 in buffer C were introduced and incubated for 5 minutes before washing off with buffer C.

For obtaining the labeling efficiency and single antigen cluster size (**Fig. 1c-1e**), we introduced 1nM P1*-Cy3B and 5nM P3*-ATTO647N imagers (See **Supplementary Table 2** for the sequences) in the imaging buffer for imaging the antibody, nanobody, and antigen positions, respectively. We first imaged ATTO647N using the 642 nm laser and then Cy3B with the 561 nm laser, each for 15000 frames at an acquisition rate of 10 Hz. (See **Supplementary Table 4** for imaging conditions).

In case the sample was used for qPAINT imaging (**Fig. 1f**), we incubated 200 pM of 20 nm grid origami with P1-extensions in buffer C for 5 minutes. Imaging was then carried out in two successive rounds. In round one, we imaged reference origami and antibody with P1*-Cy3B for 20,000 frames at a rate of 10 Hz. After washing off P1*-Cy3B imager until no signal could be observed, we then imaged the antigen positions with P3*-Cy3B imager for 15000 frames at a rate of 10 Hz.

For origami imaging (**Fig. 2**), 250 pM of origami structures carrying either ALFA-tag or CTD peptide in buffer C were introduced after the neutravidin incubation step and incubated for 5 minutes before washing off excess origamis. Similar to the single-molecule experiments, we incubated with 60 nM primary rabbit antibody against CTD before washing. We then incubated with P1-conjugated anti-rabbit secondary antibody for 30 minutes. Labeling with secondary nanobody was also performed similarly to the single-molecule experiments. Gold nanoparticles diluted 1:10 in buffer C were introduced and incubated for 5 minutes before washing with buffer C. For labeling the origami with ALFA-tag, we incubated 100 nM P1-conjugated ALFA-tag nanobody for 60 minutes in buffer C. Imaging was carried out in two rounds: one for obtaining the antibody or nanobody positions and the other for positions of antigens using P1*-Cy3B and P3*-Cy3B, respectively with a thorough buffer wash step in between. Data were acquired for 15,000 frames at 10 Hz.

Cell imaging: A549 cells (ATCC) were cultured in DMEM (Thermo Fisher Scientific, cat: 61965) supplemented with 10 % fetal bovine serum. Approximately 30,000 A549 cells were seeded into 8-well chambered coverslips (ibidi, cat: 80827) and grown overnight. On the next day, cell medium was aspirated, and cells were fixed with 4% PFA in PBS (pre-warmed to 37 °C) for 15 min at room temperature. After fixation, cells were washed three times with PBS. Then, cells were permeabilized with 0.25 % Triton-X-100 for 5 min and afterwards blocked in blocking buffer (3 % BSA in PBS) for 30 min. Primary antibody against CTD (1:200) and anti-rabbit IgG nanobody (25 nM) were incubated with the cells in blocking buffer for 1 h at room temperature. Unbound antibodies were washed away three times with PBS and then with buffer C for 10 min. Finally, buffer C was added containing 100 pM P1 Cy3B imager strands.

Cluster analysis. Initial data analysis such as localizing individual blinking events from raw data and drift correction (first redundant cross-correlation and then undrifting from picked molecules) were done using 'Localize' and 'Render' modules of the 'Picasso' software[3]. For obtaining the average cluster size corresponding to single antigens, we picked all the P1 sites (antibody/nanobody/direct extension) that showed a signal in the P3 channel (i.e. antigen positions). The picked individual P1 sites were then overlaid by their center-of-mass as the reference point using the 'Average' module of 'Picasso'. The resulting data was then displayed using the 'Render' module. For obtaining the histogram of localizations for the averaged cluster size, we projected the localizations onto one axis and performed a cross-sectional histogram analysis using OriginPro 2019b (OriginLab Corporation).

We obtained average clusters of origami for **Figure 2** in the following way: Origami data was first reconstructed by using 'Localize' module of 'Picasso'. The output data was then drift-corrected first by redundant cross correlation and then using the individual sites on the origami using 'Render's' pick module. For obtaining the average, we picked origami with 12 binding sites in the P3 channel and corresponding origami in the P1 channel. Using the 'Average' module of Picasso software, we obtained sum structures of picked origami from the P3 channel. To sum up P1 origami with the same transformation as P3 channel data, we first compared individual P3 origami structures with its average one and obtained the geometrical transformation (rotation and translation) for each of the

structures. We then applied each transformation of P3 to its corresponding P1 structures and then overlaid them to obtain the sum image.

Labeling efficiency analysis. Efficiency of labeling for the antigen (Digoxigenin, 1×CTD, and 5×CTD) or ALFA-Tag by corresponding binders was evaluated by measuring the signal from the antibody or nanobody colocalized with the signal from the antigens/ALFA-Tag. In **Figure 1b** and **c**, P3*-ATTO647N data corresponds to the position of antigen or ALFA-Tag and P1*-Cy3B data to the antibody or nanobody. We first listed the individual spots, representing single molecules, in the P3 channel that showed a minimum of 25 localizations in a 100 nm diameter pick. We then looked for corresponding localizations in the P1 channel in each of the picks. If we detected more than 25 localizations, we defined this as co-localization. The labeling efficiency was then calculated according to: $LE = CL_{P3\rightarrow P1}/P3_{total}$ with LE = Labeling Efficiency and $CL_{P3\rightarrow P1}$ = Co-localization of P1 signal at P3 signal location.

qPAINT analysis. To count the total number of docking strands present on each antigen (carried by secondary antibodies bound to primary antibodies) we used qPAINT[5]. We immobilized DNA origami structures carrying twelve docking strands with the same sequence as the secondary antibody or ALFA-tag nanobody. We then analyzed the binding frequency of the imager strands on the origami nanostructures, used this as calibration to compute absolute number of strands. We then performed binding frequency analysis for the antibody and nanobody case to obtain absolute number of docking strands per label.

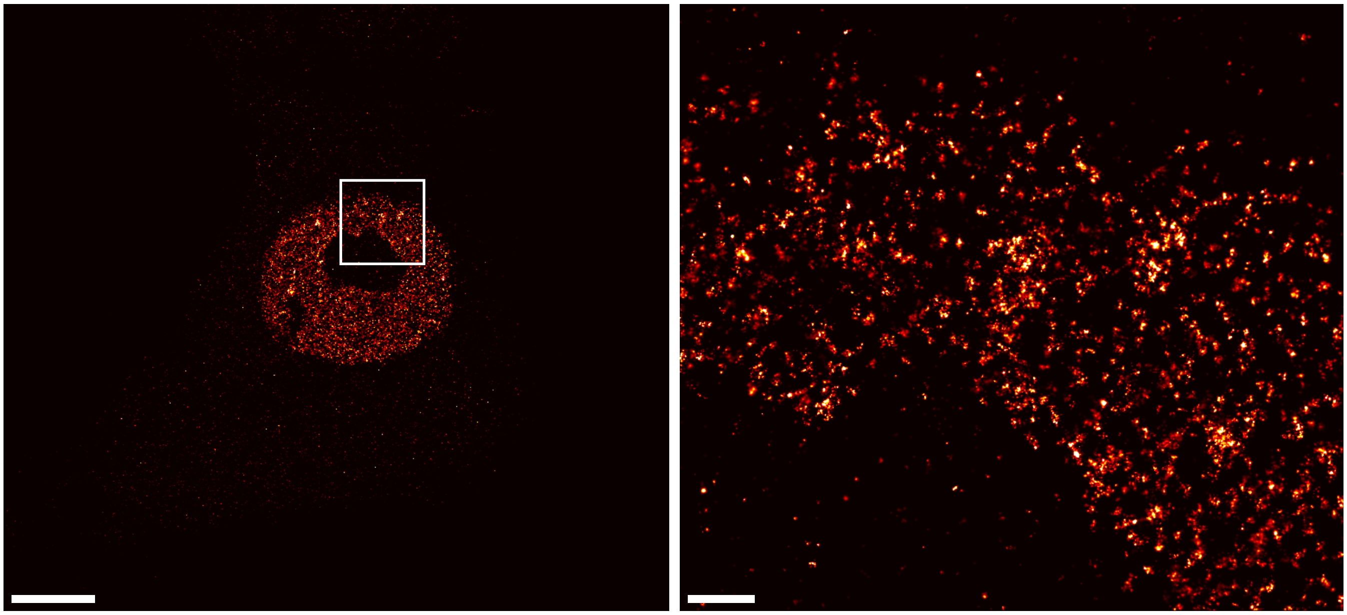

Figure S1 | DNA-PAINT imaging of the CTD domain of RNA polymerase using primary and DNA-conjugated secondary nanobodies in HeLa cells. Left: Overview image showing specific signal in the cell nucleus. Right: Zoom-in of the highlighted area in the left figure showing nanoclusters of RNA-polymerase. This data demonstrates that the chosen CTD antibody shows specific signal in the nucleus with qualitatively sufficient labeling efficiency. Scale bars: 5 µM (left), 500 nm (right).

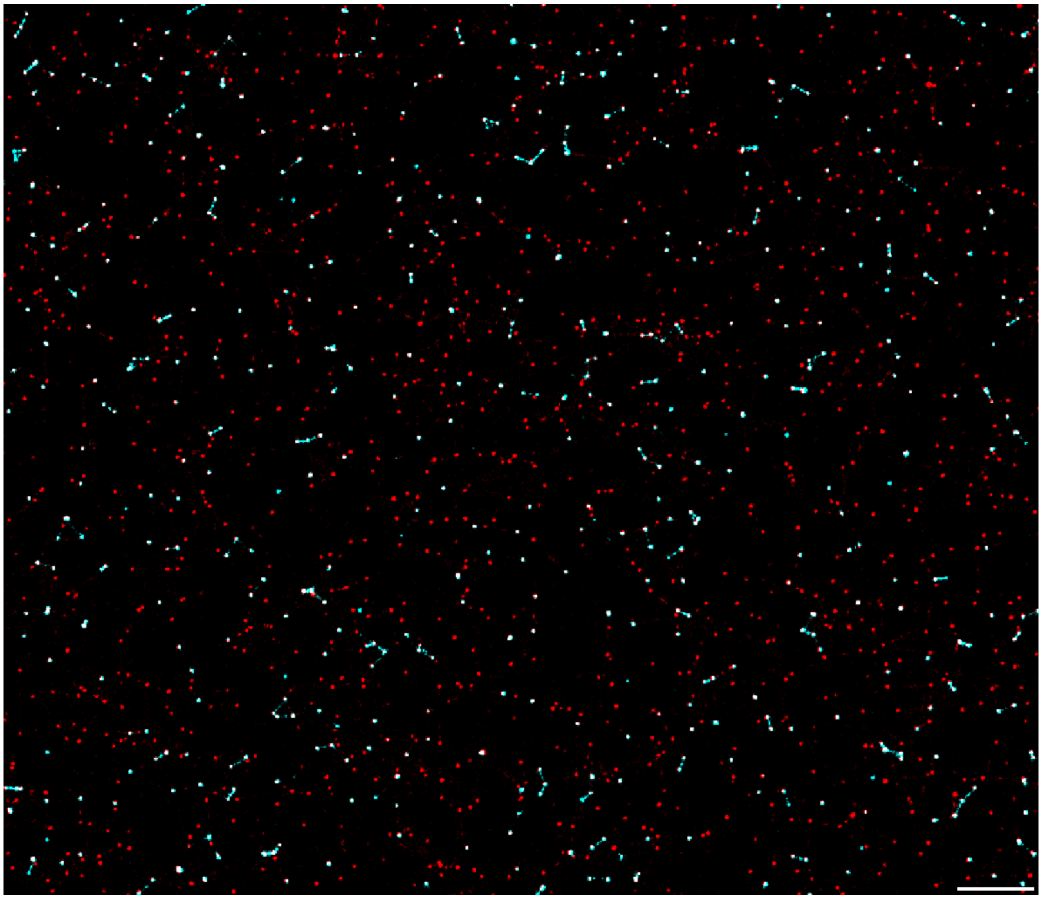

Figure S2 | Overview image of DIG single-molecule experiments. Antigen (DIG) position acquired using ATTO647N-labeled P3* imager strands (red) and antibody position obtained with Cy3B-labeled P1* imager strands (cyan). Scale bar: 1 µm

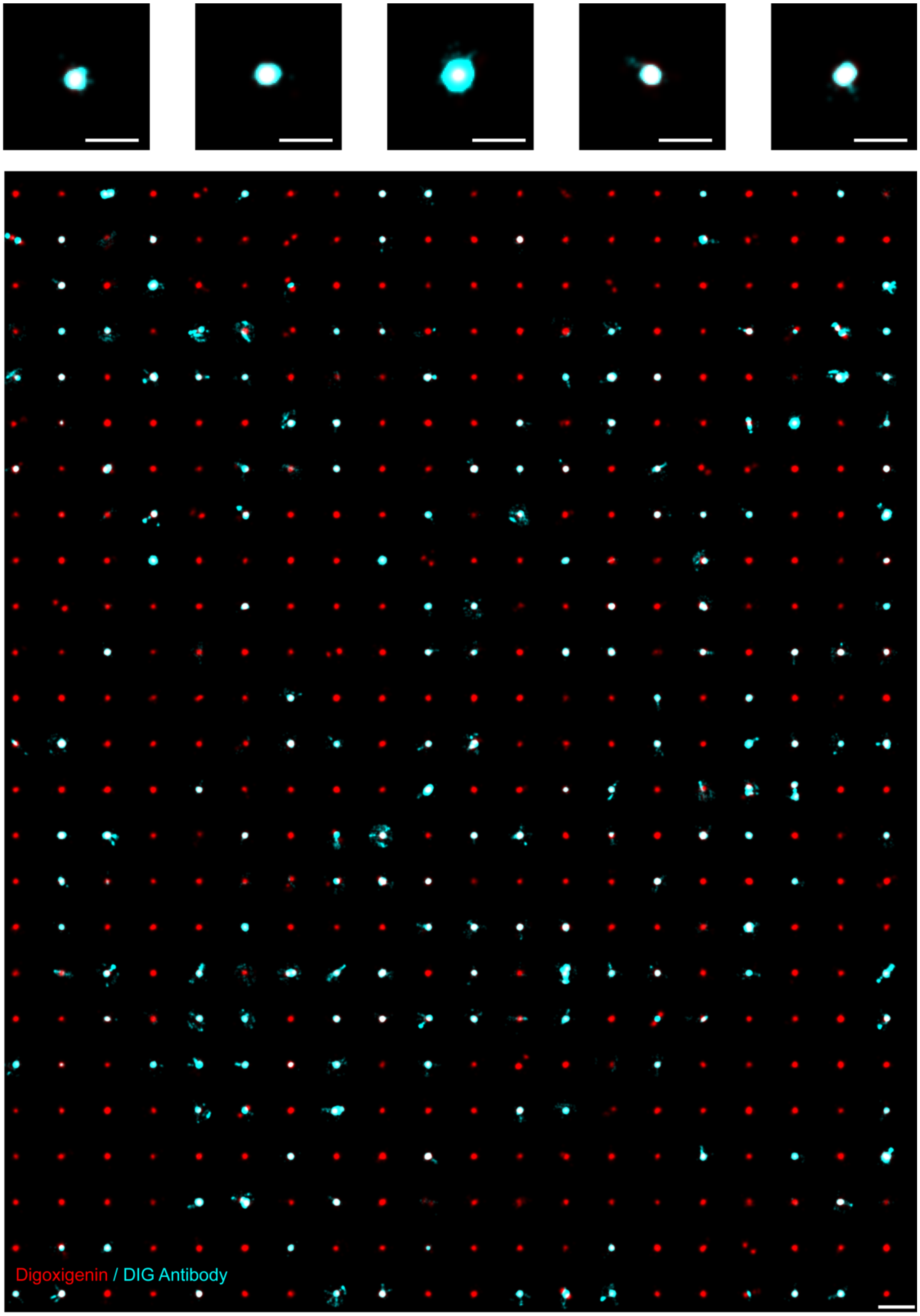

Figure S3 | Single DIG sites selected for sum image from single-molecule experiments. Five exemplary selected sites (top). Overview of individual selected sites for sum image (bottom). Antigen (DIG) position was imaged with ATTO647N-labeled P3* imager strands (red). Secondary antibody position was imaged with Cy3B-labeled P1* imager strands (cyan). Scale bars: 100 nm (top), 200 nm (bottom).

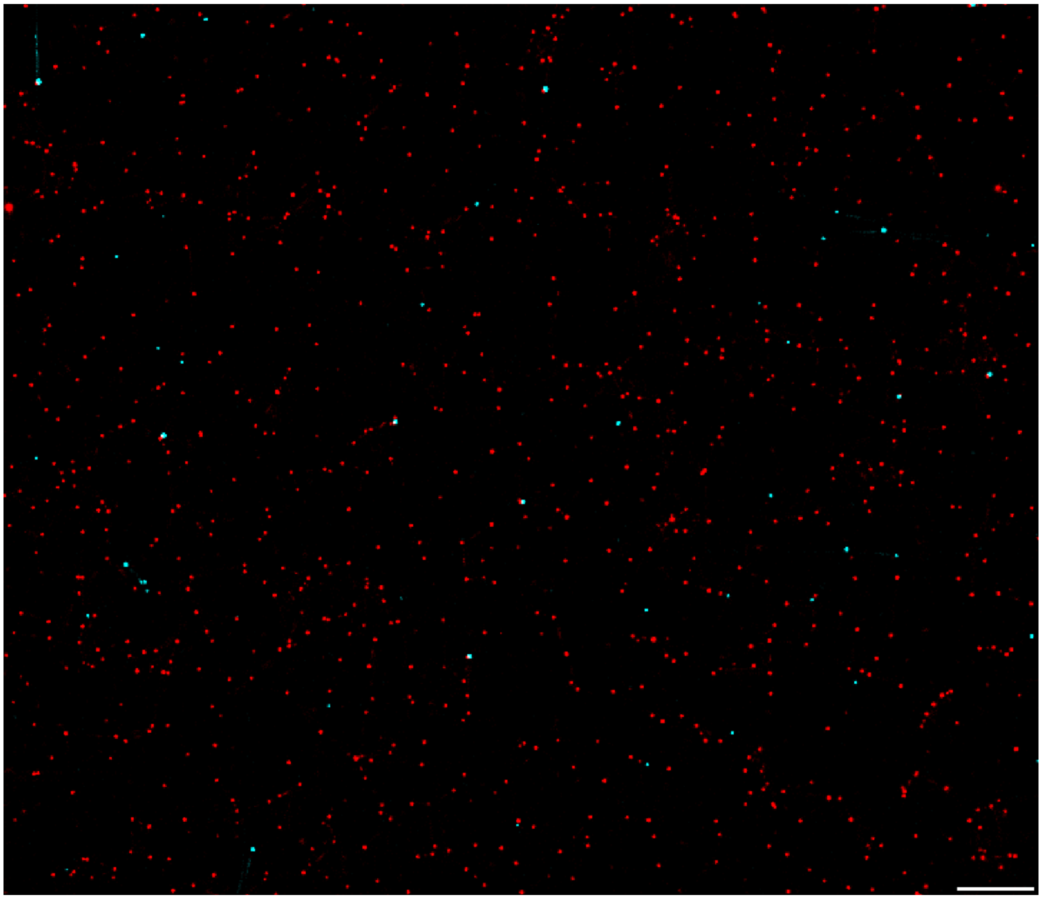

Figure S4 | Overview image of 1xCTD single-molecule experiments. Antigen (1xCTD) position acquired using ATTO647N-labeled P3* imager strands (red) and antibody position obtained with Cy3B-labeled P1* imager strands (cyan). Scale bar: 1 µm

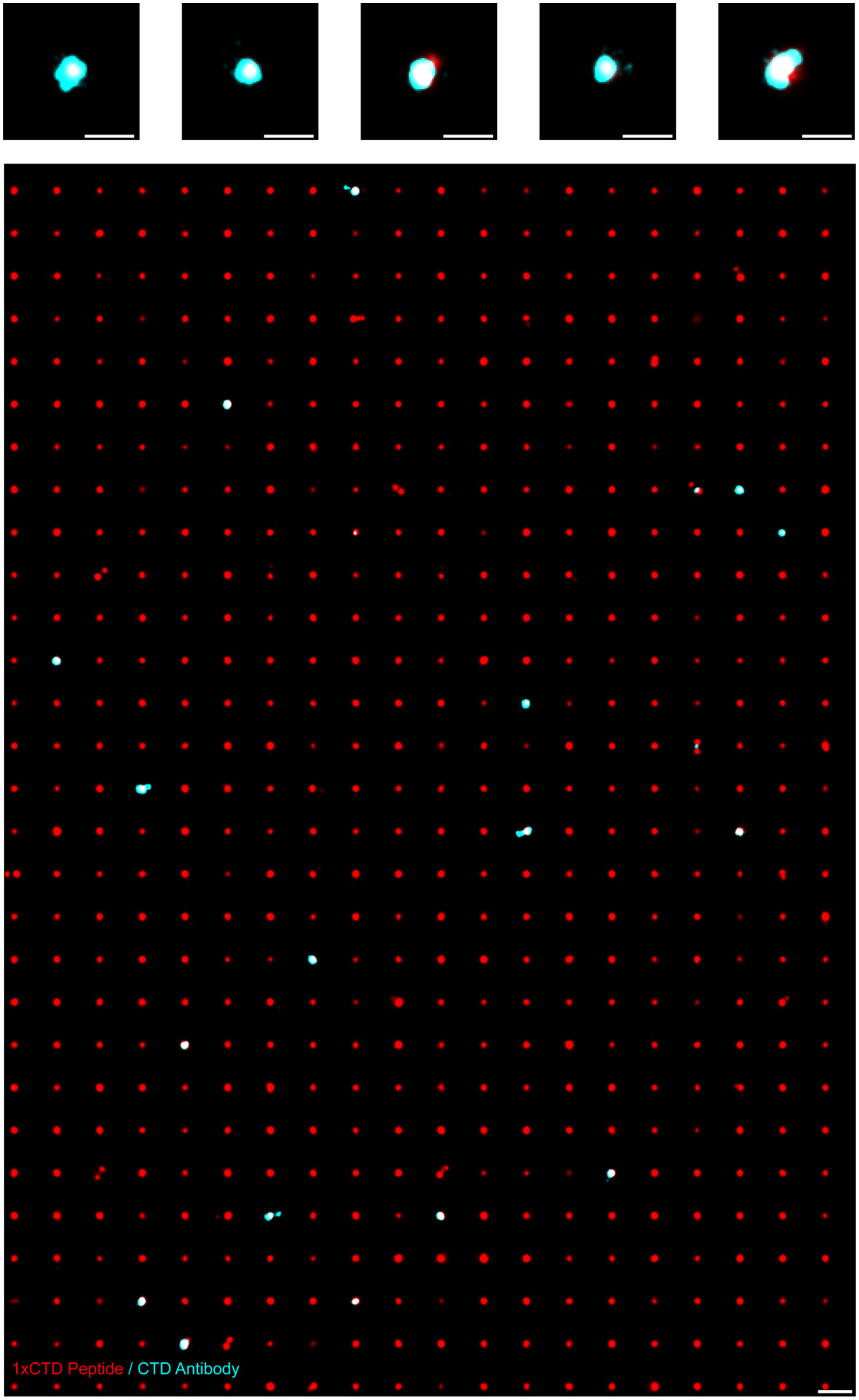

Figure S5 | Single 1xCTD sites selected for sum image and probe-based resolution estimate from single-molecule experiments. Five exemplary selected sites (top). Overview of individual selected sites for sum image (bottom). Antigen (1xCTD) position was imaged with ATTO647N-labeled P3*

imager strands (red). Secondary antibody position was imaged with Cy3B-labeled P1* imager strands (cyan). Scale bars: 100 nm (top), 200 nm (bottom).

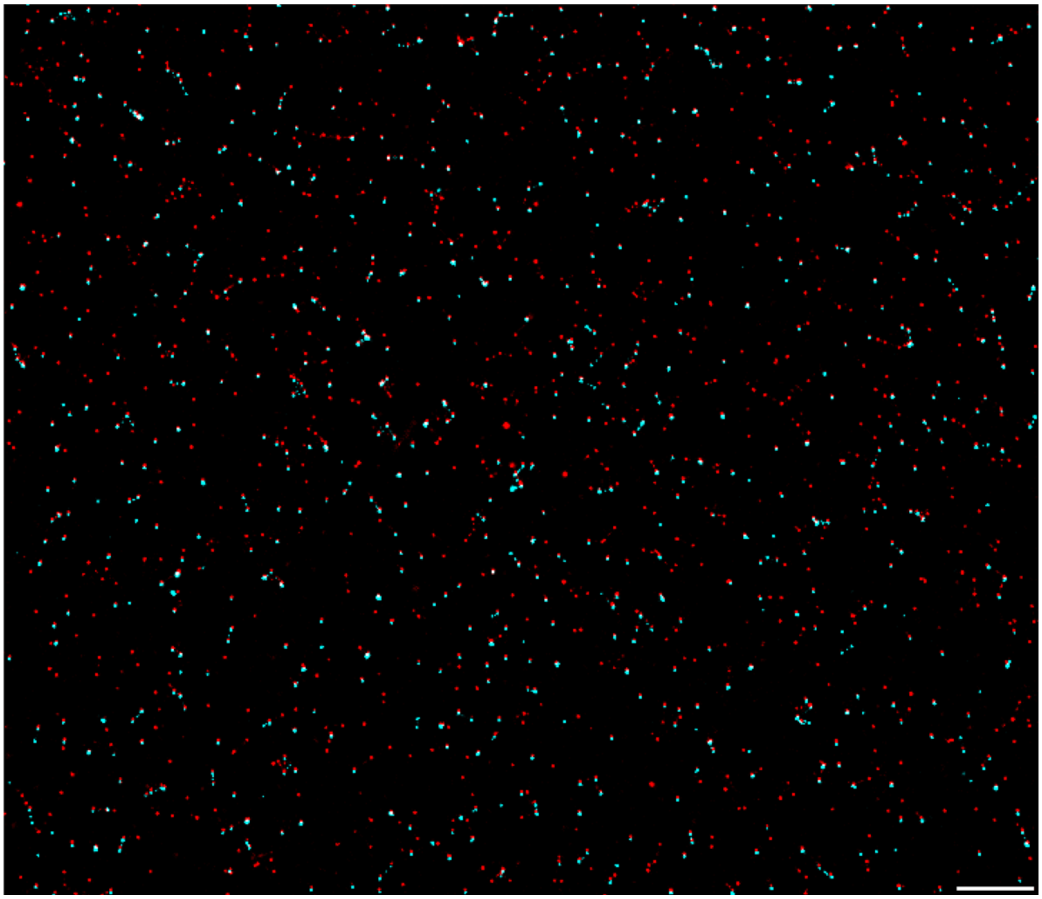

Figure S6 | Overview image of 5xCTD repeat single-molecule experiments. Antigen (5xCTD) position acquired using ATTO647N-labeled P3* imager strands (red) and antibody position obtained with Cy3B-labeled P1* imager strands (cyan). Scale bar: 1 µm

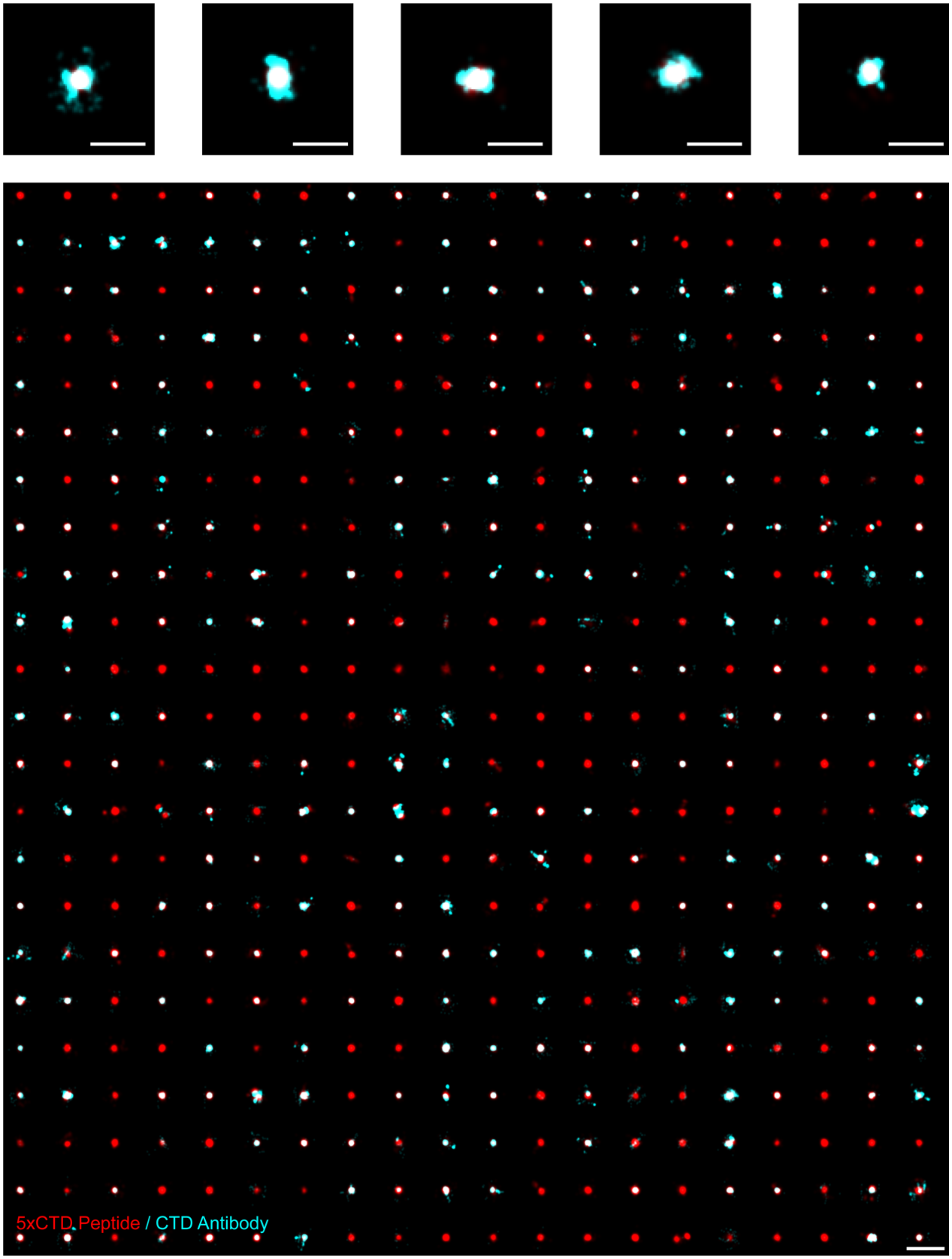

Figure S7 | Single 5xCTD repeat sites selected for sum image and probe-based resolution estimate from single-molecule experiments. Five exemplary selected sites (top). Overview of individual selected sites for sum image (bottom). Antigen (5xCTD) position was imaged with ATTO647N-labeled P3* imager strands (red). Secondary antibody position was imaged with Cy3B-labeled P1* imager strands (cyan). Scale bars: 100 nm (top), 200 nm (bottom).

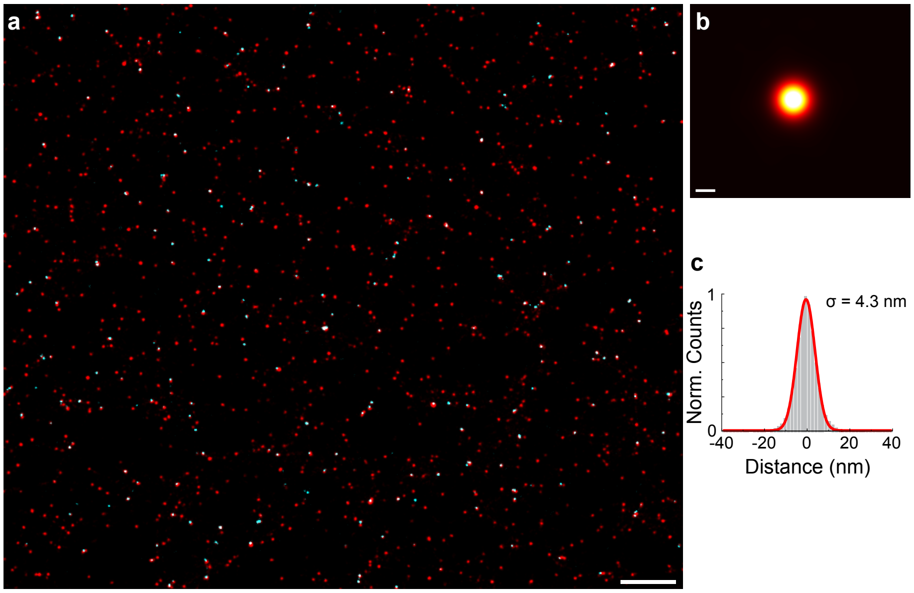

Figure S8 | Overview image of 1xCTD repeat single-molecule experiments imaged with primary antibody and secondary nanobody. (**a**) Antigen (1xCTD) position acquired using ATTO647N-labeled P3* imager strands (red) and secondary nanobody that bound to primary CTD antibody position obtained with Cy3B-labeled P1* imager strands (cyan). (**b**) Center-of-mass-aligned localizations of single molecules and (**c**) corresponding cross-sectional histograms for antibody-secondary nanobody (n = 599, solid line is Gaussian fit). Scale bar: 1 µm (**a**), 10 nm (**b**)

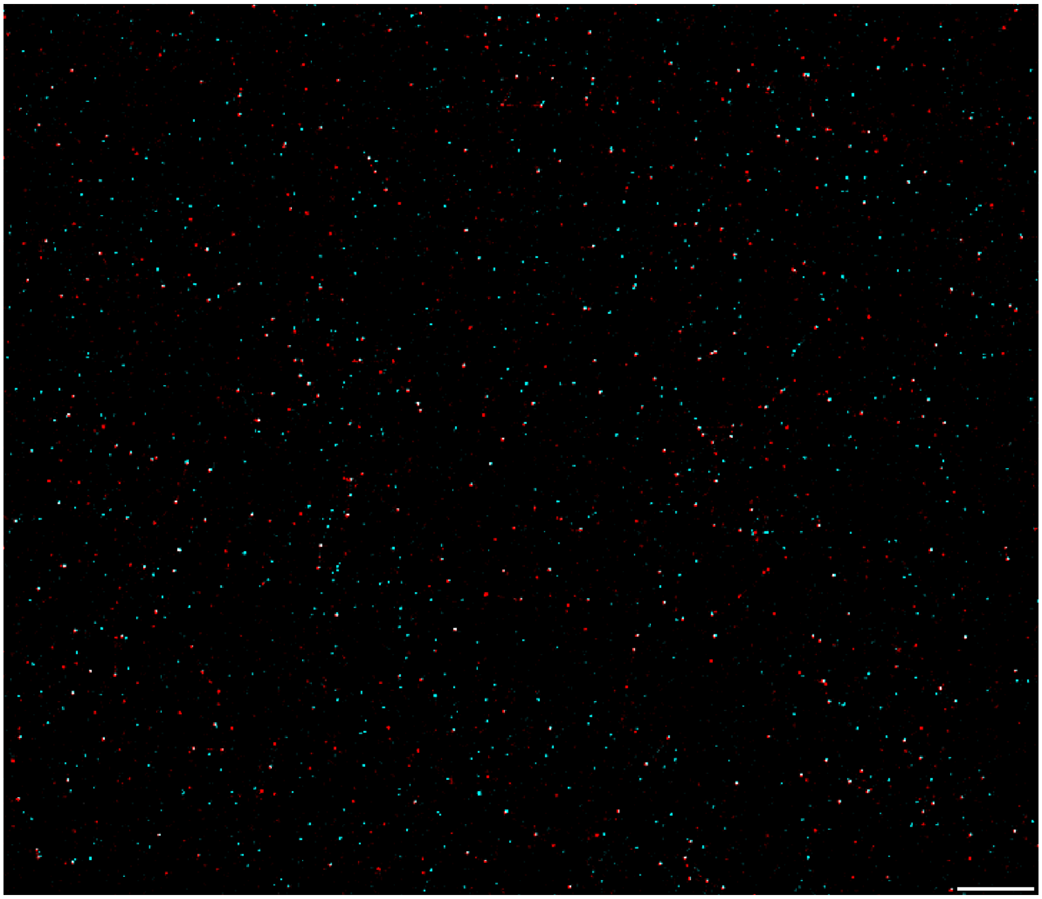

Figure S9 | Overview image of ALFA-tag based single-molecule experiments. Antigen (ALFA-tag) position acquired using ATTO647N-labeled P3* imager strands (red) and nanobody position obtained with Cy3B-labeled P1* imager strands (cyan). Scale bar: 1 µm

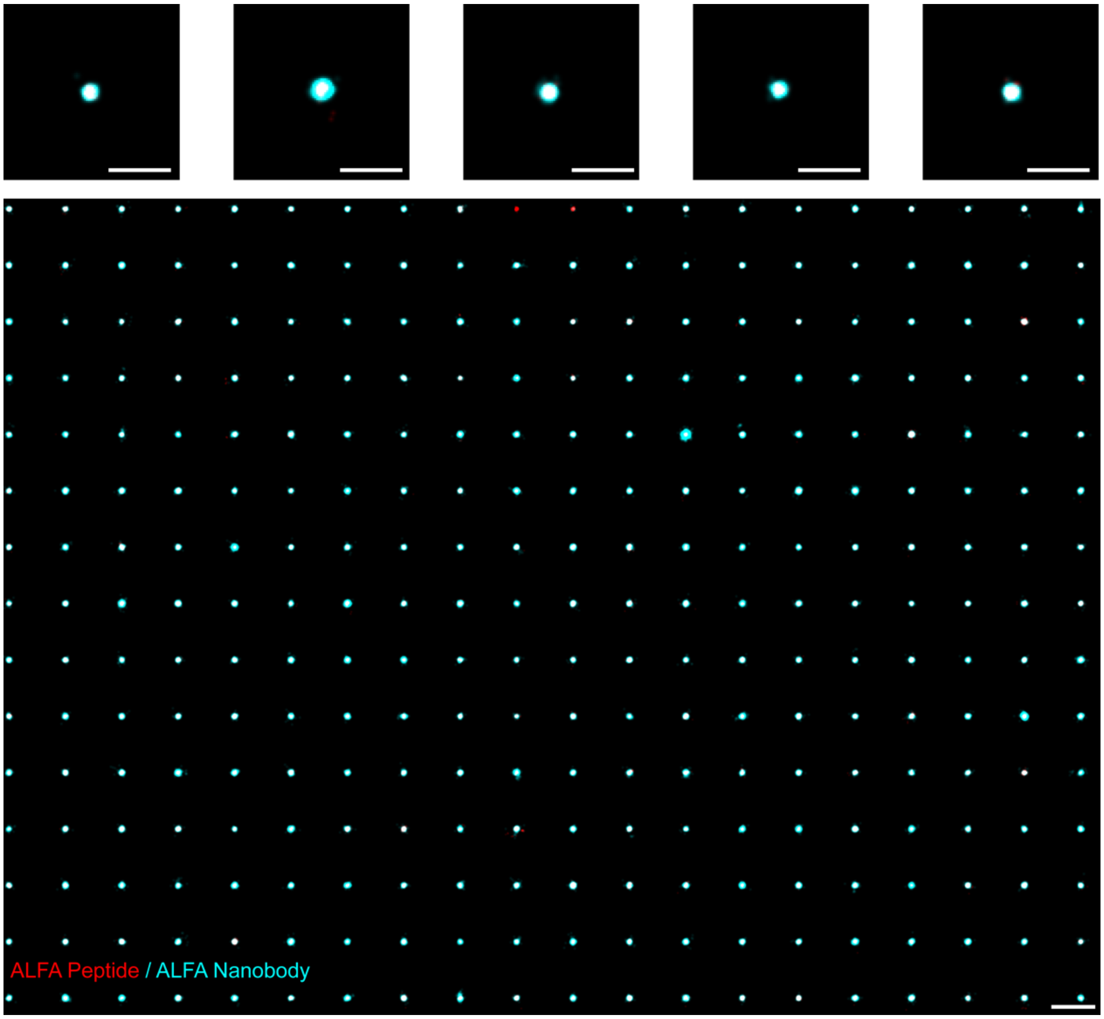

Figure S10 | Single ALFA-tag sites selected for sum image and probe-based resolution estimate from single-molecule experiments. Five exemplary selected sites (top). Overview of individual selected sites for sum image (bottom). Antigen (ALFA-tag) position was imaged with ATTO647N-labeled P3* imager strands (red). Nanobody position was imaged with Cy3B-labeled P1* imager strands (cyan). Scale bars: 100 nm (top), 200 nm (bottom).

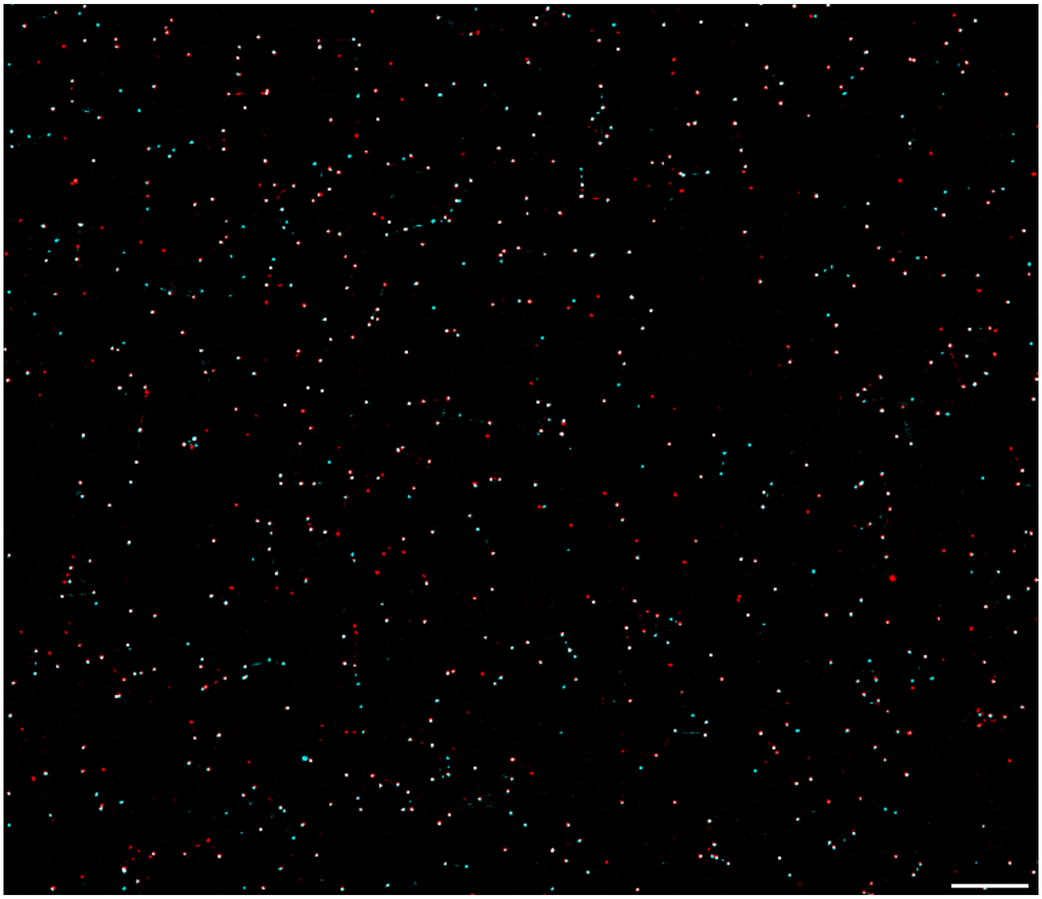

Figure S11 | Overview image of directly extended DNA-PAINT docking strand single molecule experiments. True target position acquired using ATTO647N-labeled P3* imager strands (red) and position of direct extension on the same strand obtained with Cy3B-labeled P1* imager strands (cyan). Scale bar: 1 µm

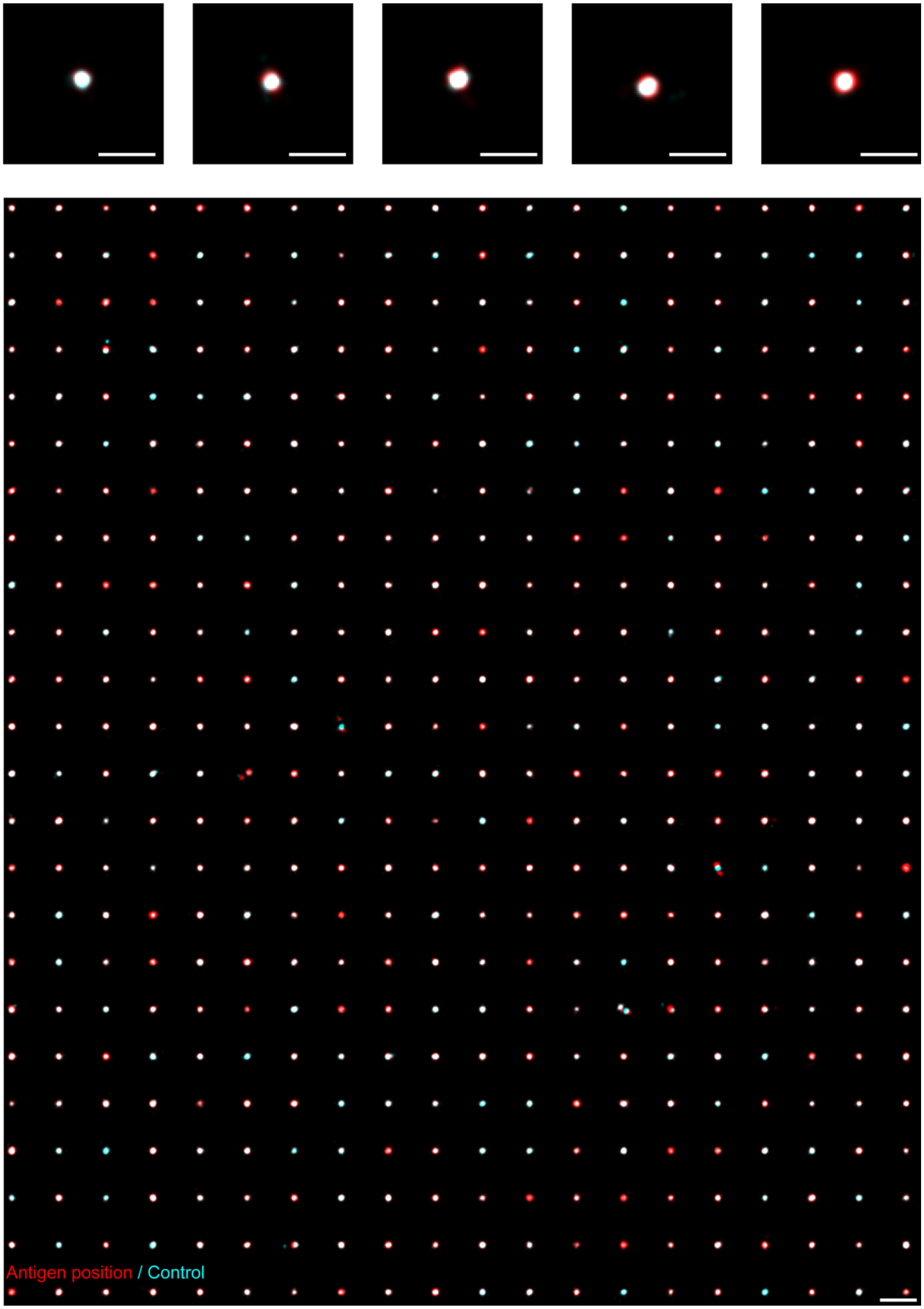

Figure S12 | Single directly extended sites selected for sum image and probe-based resolution estimate from single molecule experiments. Five exemplary selected sites (top). Overview of individual selected sites for sum image (bottom). True target position was imaged with ATTO647N-labeled P3* imager strands (red). Secondary antibody signal was imaged with Cy3B-labeled P1* imager strands (cyan). Scale bars: 100 nm (top), 200 nm (bottom).

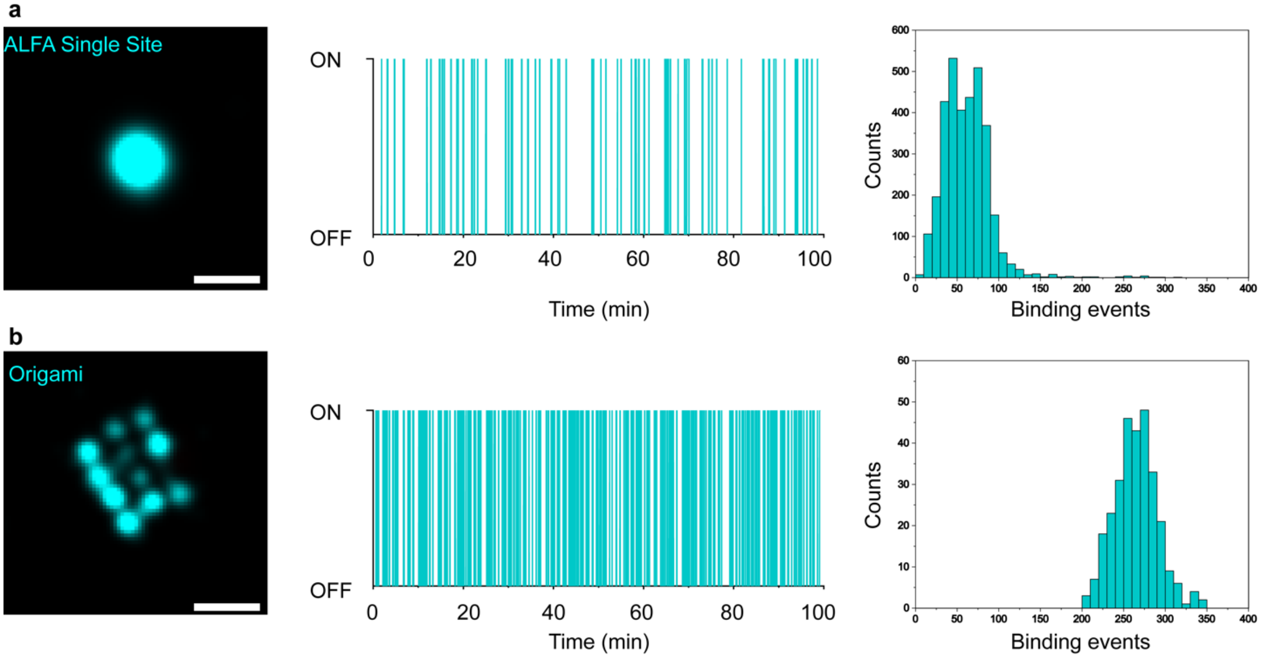

Figure S13 | qPAINT comparison of a single ALFA-tag site with 20-nm-grid DNA origami calibration standard. (**a**) Single ALFA-tag site (left). Repetitive visits of DNA-PAINT imager strands to their complementary target site at the ALFA-tag nanobody over the whole course of image acquisition (middle). Histogram of imager binding events for the picked single sites (right). (**b**) Corresponding data for DNA origami. Scale bars: 50 nm.

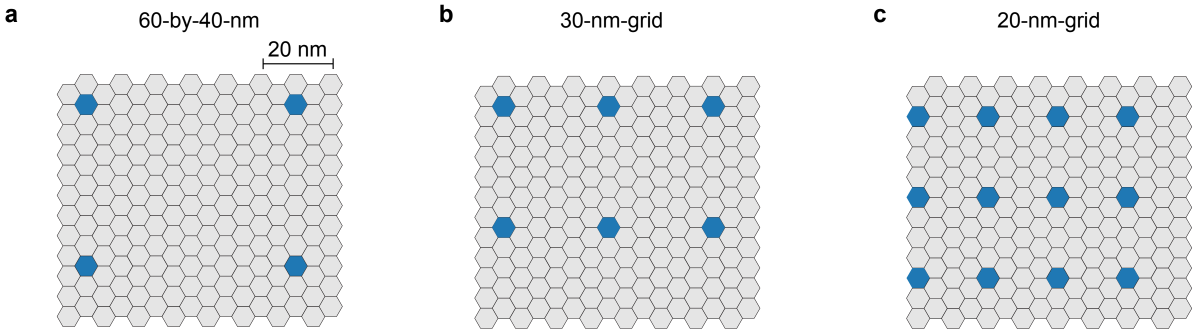

Figure S14 | Overview of DNA origami structures. (**a**) 60-by-40-nm DNA origami design (blue hexagons are antigen positions). (**b**) Corresponding 30-nm-grid structure. (**c**) Corresponding 20-nm-grid structure.

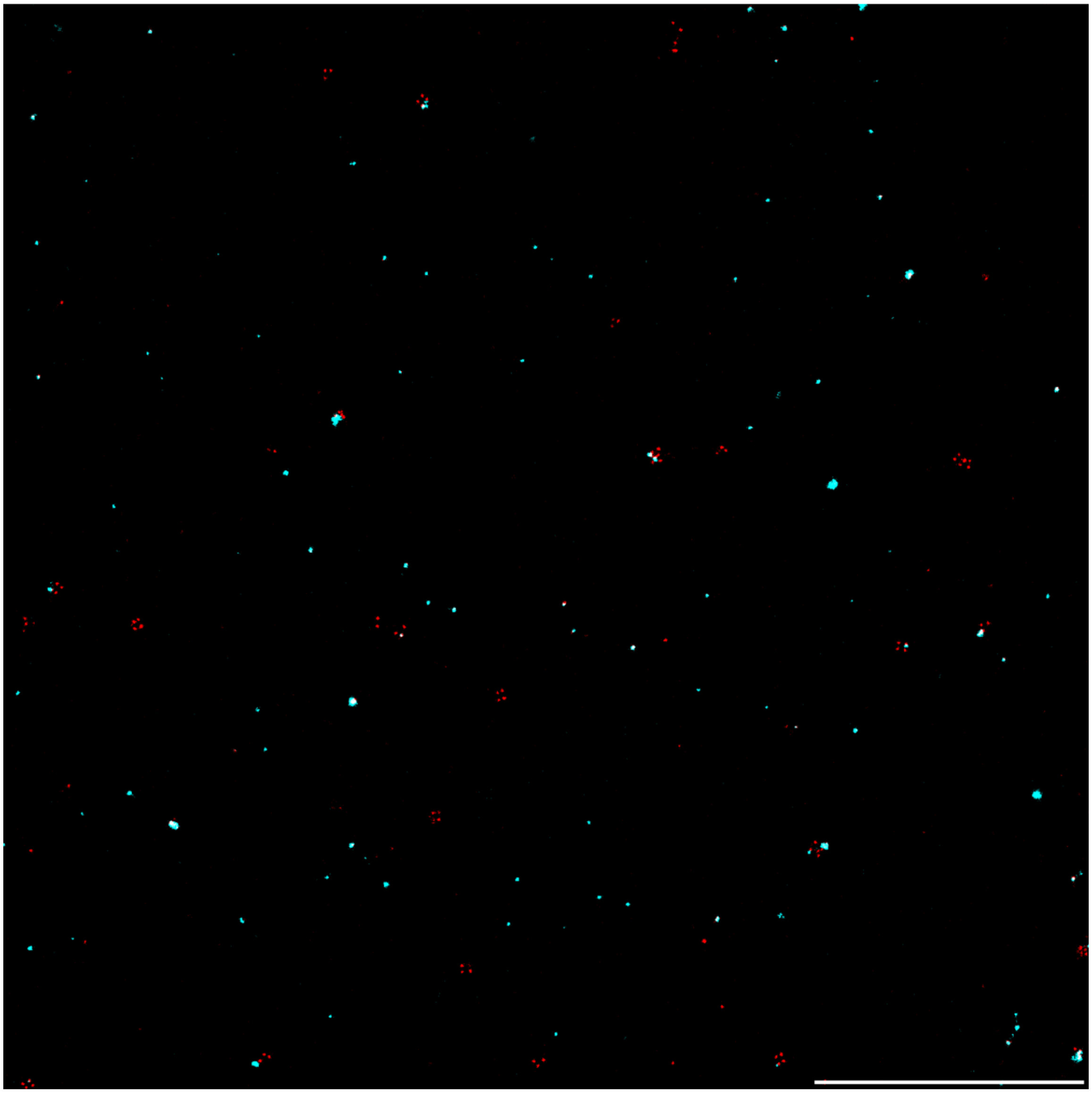

Figure S15 | Overview image of 1xCTD nanoclusters templated at 40 and 60 nm distances. Antigen (1xCTD) position was imaged with Cy3B-labeled P3* imager strands (red) and the secondary antibody was imaged with Cy3B-labeled P1* imager strands (cyan) using Exchange-PAINT. Scalebar: 2 µm

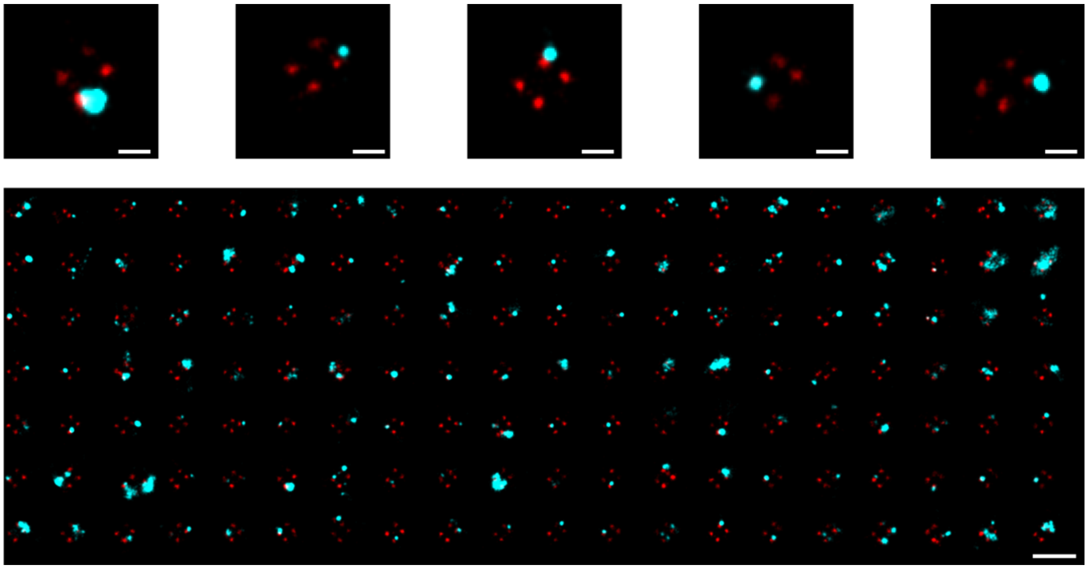

Figure S16 | Selected 1x CTD nanoclusters templated at 40x60nm distances for probe-based resolution estimate and labeling shadow determination. Five exemplary nanoclusters (top). Selected nanocluster for labeling shadow estimation (bottom). Antigen (1xCTD) position was imaged with Cy3B-labeled P3* imager strands (red) and secondary antibody signal was imaged with Cy3B-labeled P1* imager strands (cyan) using Exchange-PAINT. Scale bars: 50 nm (top), 200 nm (bottom)

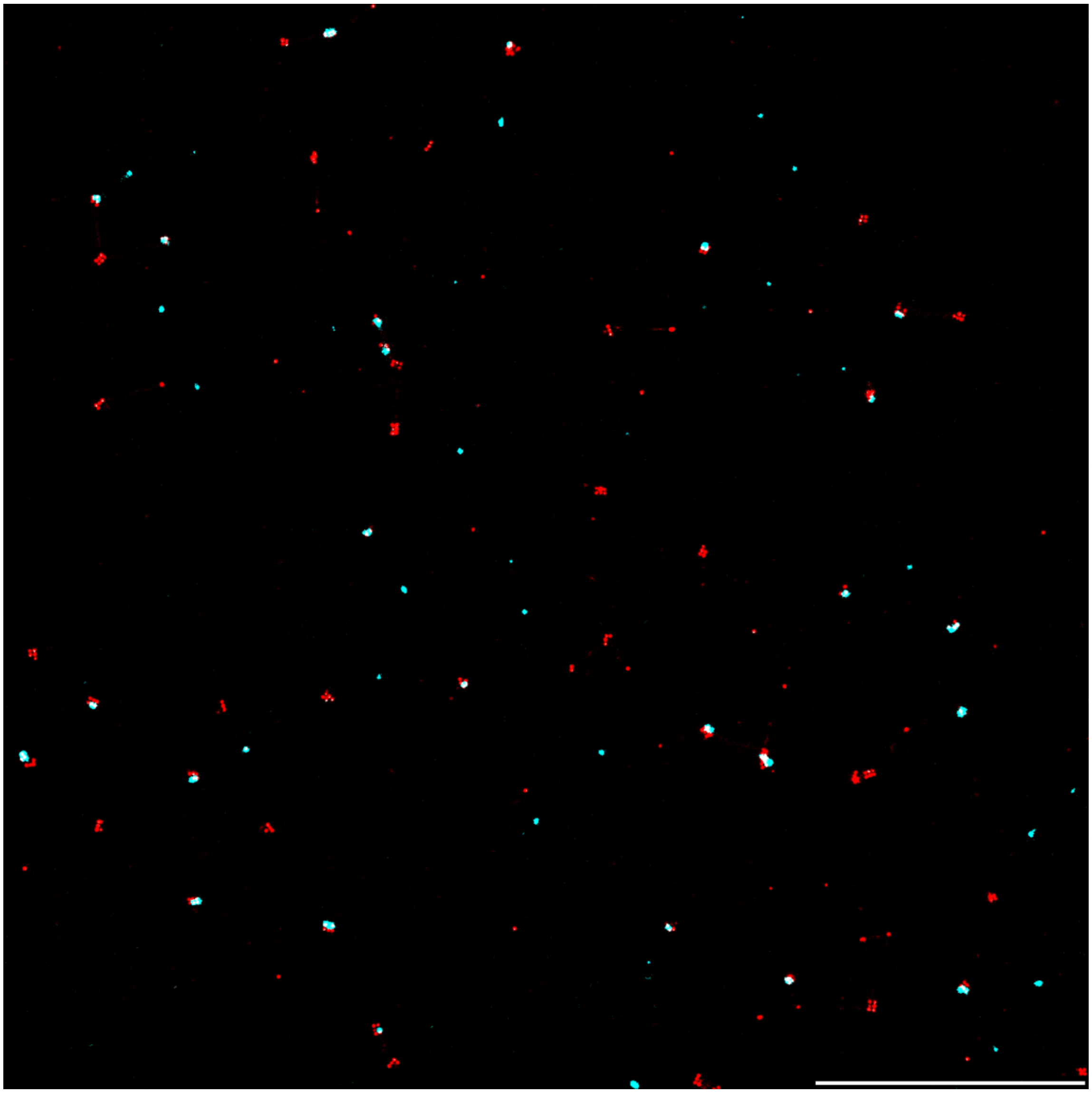

Figure S17 | Overview image of 1xCTD nanoclusters templated at 30 nm distances. Antigen (1xCTD) position was imaged with Cy3B-labeled P3* imager strands (red) and the secondary antibody was imaged with Cy3B-labeled P1* imager strands (cyan) using Exchange-PAINT. Scale bar: 2 µm

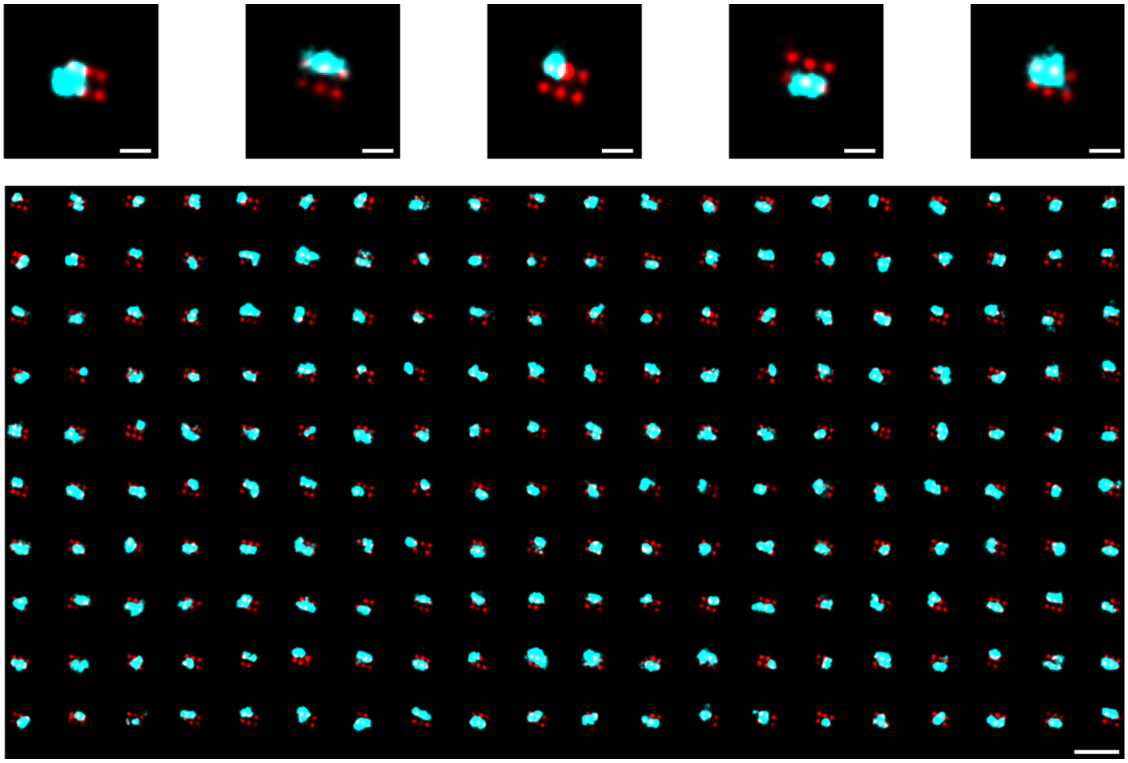

Figure S18 | Selected 1x CTD nanoclusters templated at 30 nm distances for probe-based resolution estimate and labeling shadow determination. Five exemplary nanoclusters (top). Selected nanocluster for labeling shadow estimation (bottom). Antigen (1xCTD) position was imaged with Cy3B-labeled P3* imager strands (red) and secondary antibody signal was imaged with Cy3B-labeled P1* imager strands (cyan) using Exchange-PAINT. Scale bars: top 50 nm, bottom 200 nm

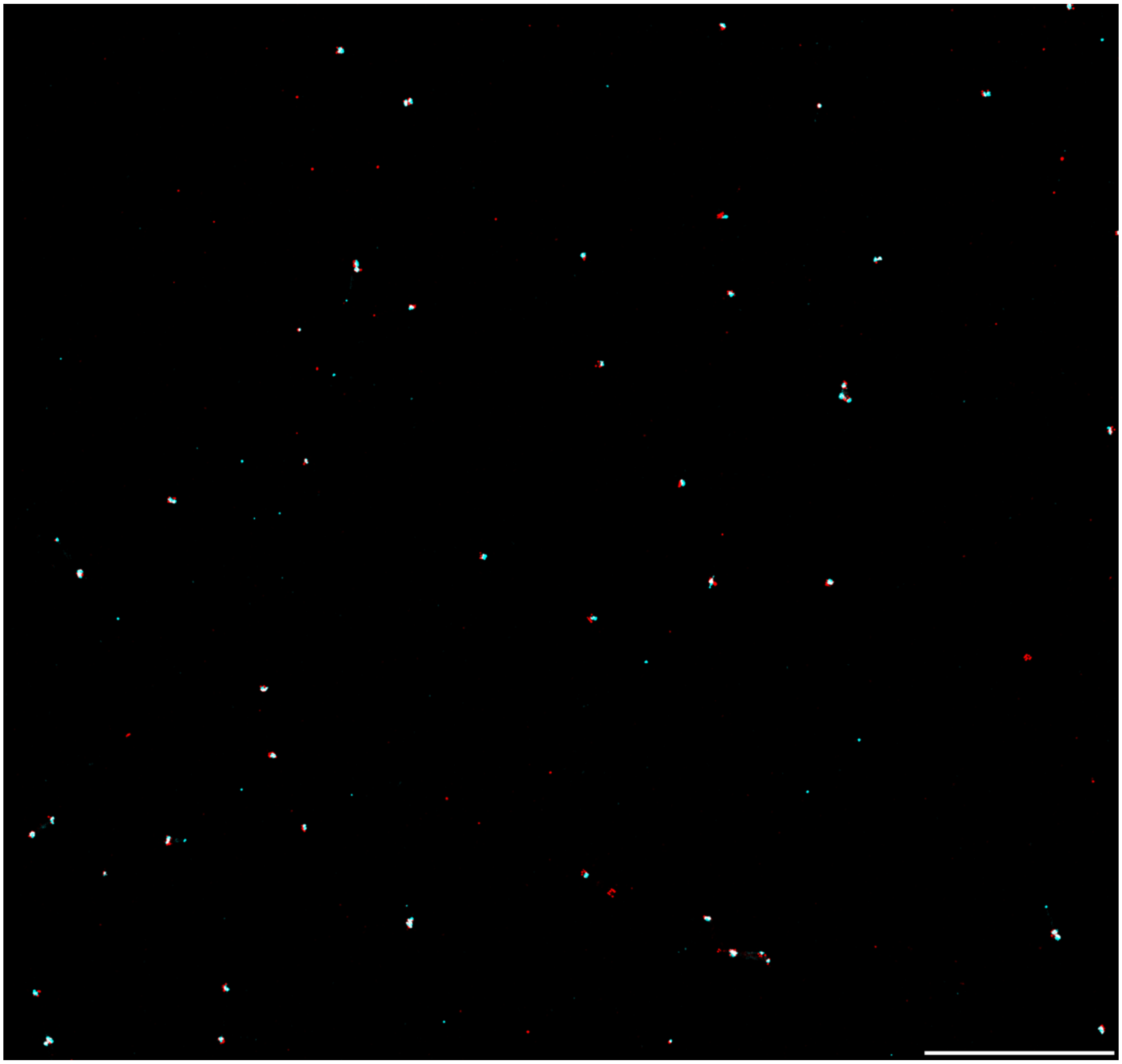

Figure S19 | Overview image of 1xCTD nanoclusters templated at 20 nm distances. Antigen (1xCTD) position was imaged with Cy3B-labeled P3* imager strands (red) and the secondary antibody was imaged with Cy3B-labeled P1* imager strands (cyan) using Exchange-PAINT. Scale bar: 2 µm

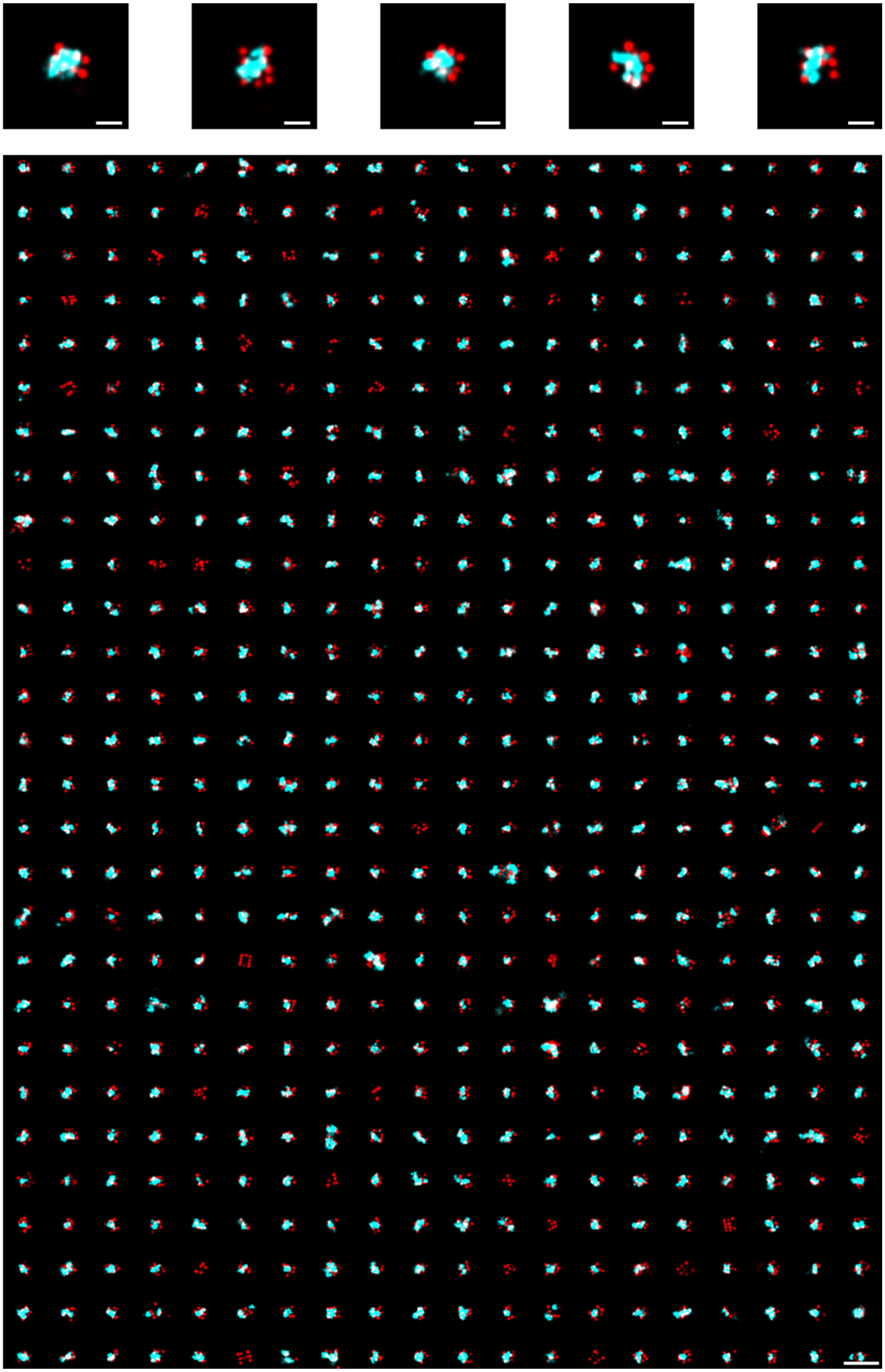

Figure S20 | Selected 1x CTD nanoclusters templated at 20 nm distances for probe-based resolution estimate and labeling shadow determination. Five exemplary nanoclusters (top). Selected nanocluster for labeling shadow estimation. Antigen (1xCTD) position was imaged with Cy3B-labeled P3* imager strands (red) and secondary antibody signal was imaged with Cy3B-labeled P1* imager strands (cyan) using Exchange-PAINT. Scale bars: 50 nm (top), 200 nm (bottom)

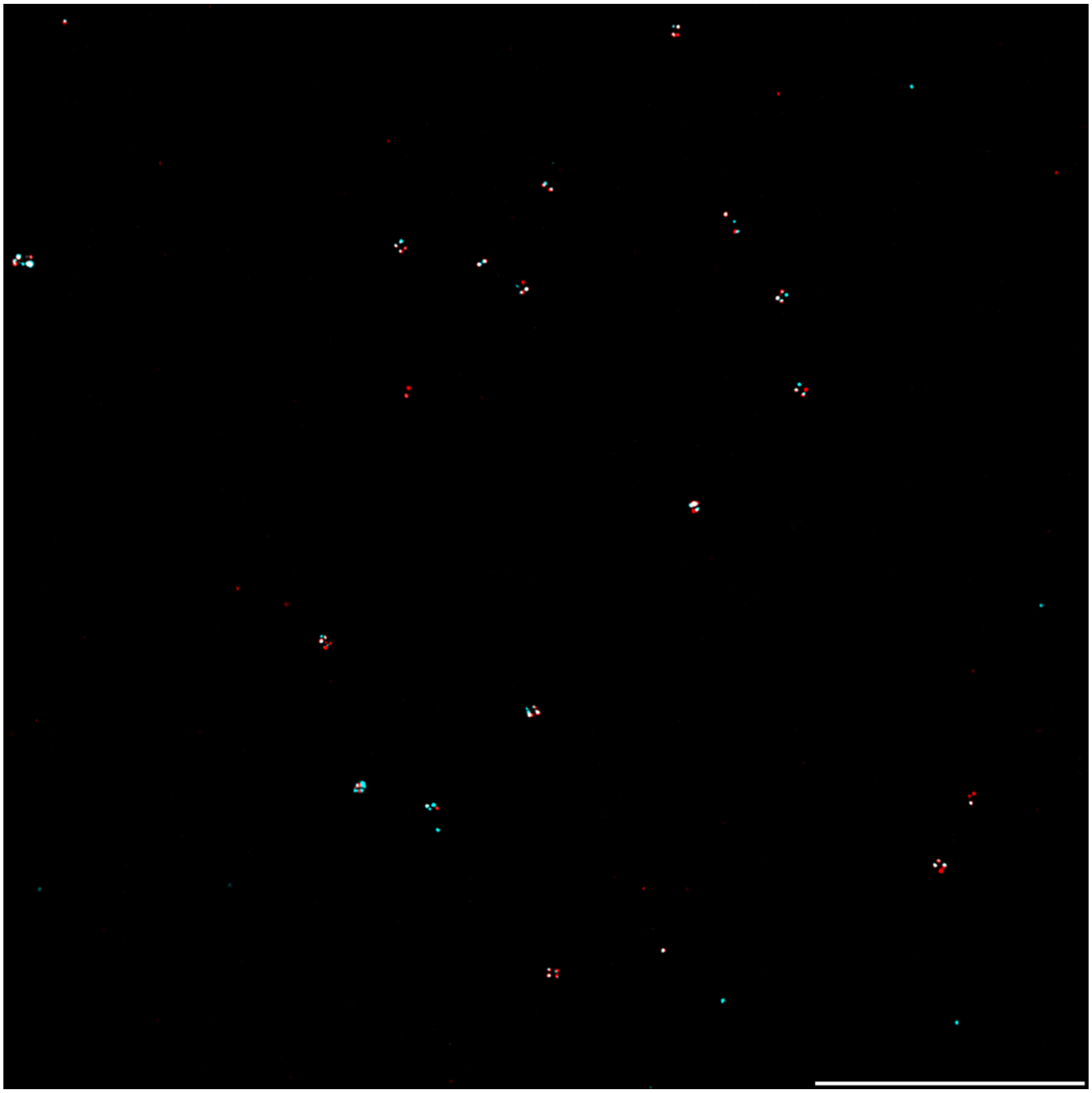

Figure S21 | Overview image of ALFA-tag nanoclusters templated at 40 and 60 nm distances. Antigen (ALFA-tag) position was imaged with Cy3B-labeled P3* imager strands (red) and the ALFA-tag nanobody was imaged with Cy3B-labeled P1* imager strands (cyan) using Exchange-PAINT. Scalebar: 2 µm

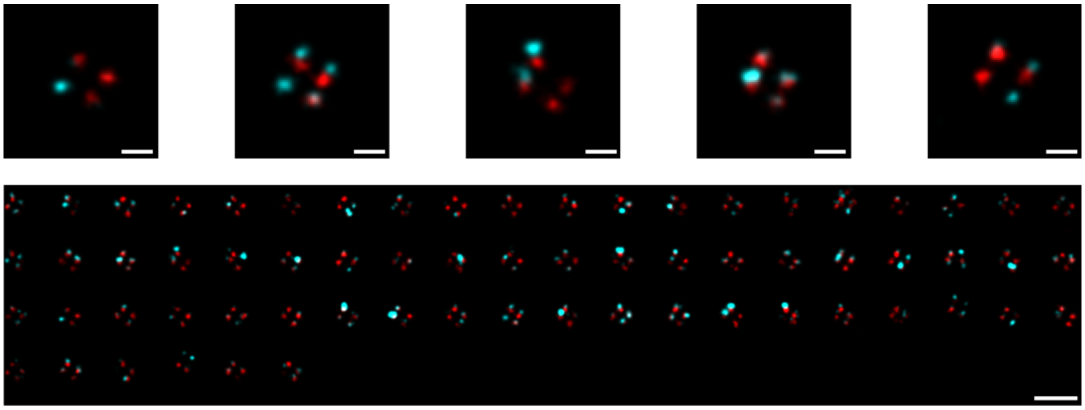

Figure S22 | Selected ALFA-tag nanoclusters templated at 40 and 60 nm distances for probe-based resolution estimate and labeling shadow determination. Five exemplary nanoclusters (top). Selected nanocluster for labeling shadow estimation (bottom). Antigen (ALFA-tag) position was imaged with Cy3B-labeled P3* imager strands (red) and ALFA-tag nanobody signal was imaged with Cy3B-labeled P1* imager strands (cyan) using Exchange-PAINT. Scale bars: 50 nm (top), 200 nm (bottom)

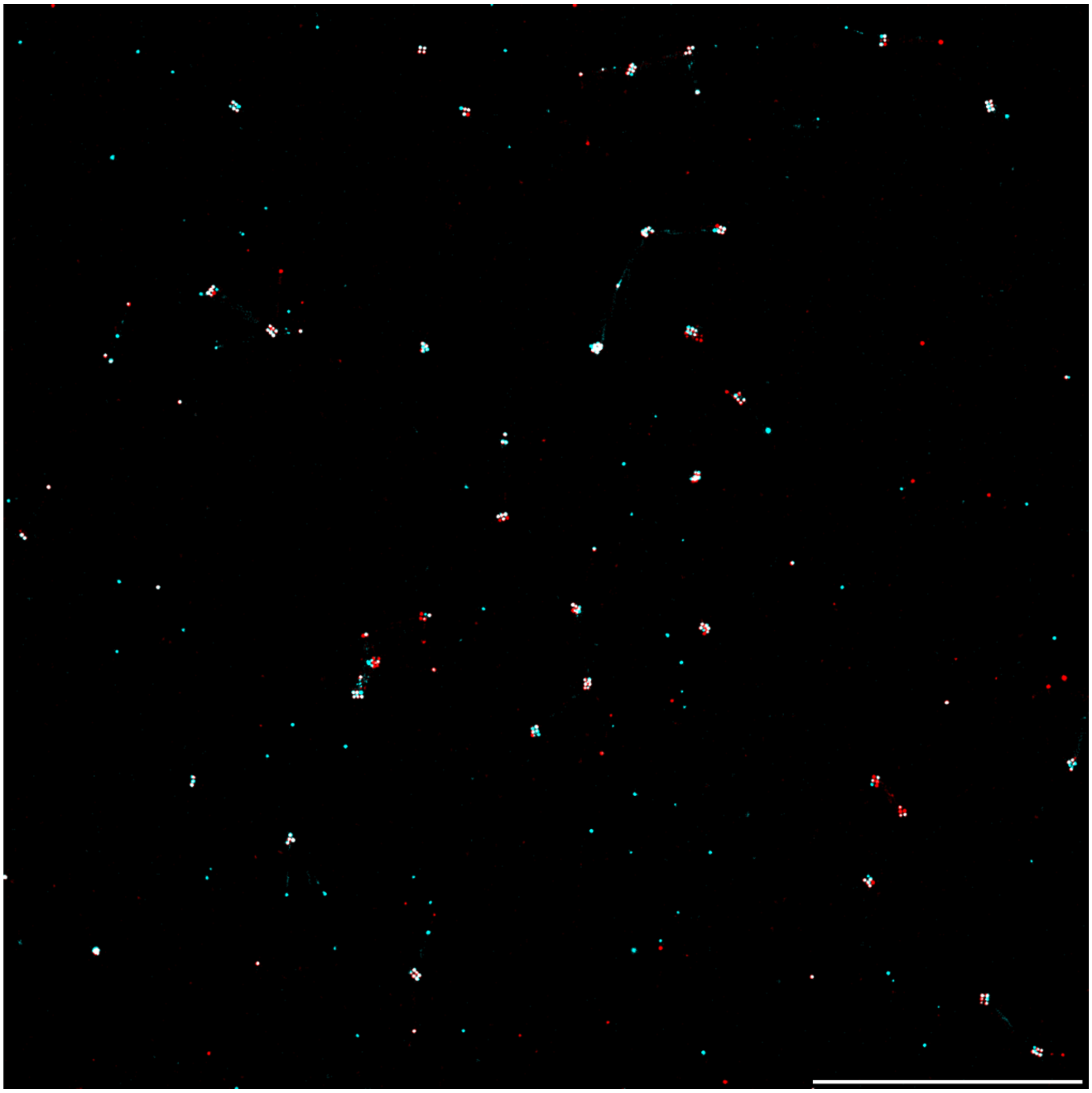

Figure S23 | Overview image of ALFA-tag nanoclusters templated at 30 nm distances. Antigen (ALFA-tag) position was imaged with Cy3B-labeled P3* imager strands (red) and the ALFA-tag nanobody was imaged with Cy3B-labeled P1* imager strands (cyan) using Exchange-PAINT. Scale bar: 2 µm

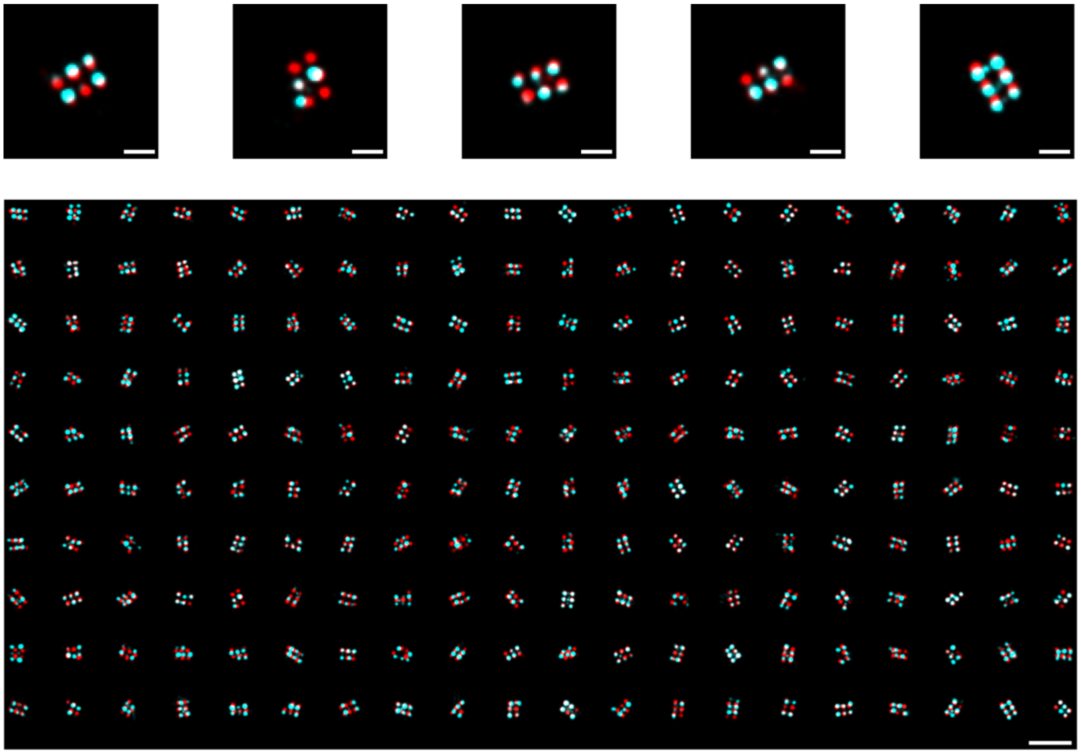

Figure S24 | Selected ALFA-tag nanoclusters templated at 30 nm distances for probe-based resolution estimate and labeling shadow determination. Five exemplary nanoclusters (top). Selected nanocluster for labeling shadow estimation (bottom). Antigen (ALFA-tag) position was imaged with Cy3B-labeled P3* imager strands (red) and ALFA-tag nanobody signal was imaged with Cy3B-labeled P1* imager strands (cyan) using Exchange-PAINT. Scale bars: 50 nm (top), 200 nm (bottom)

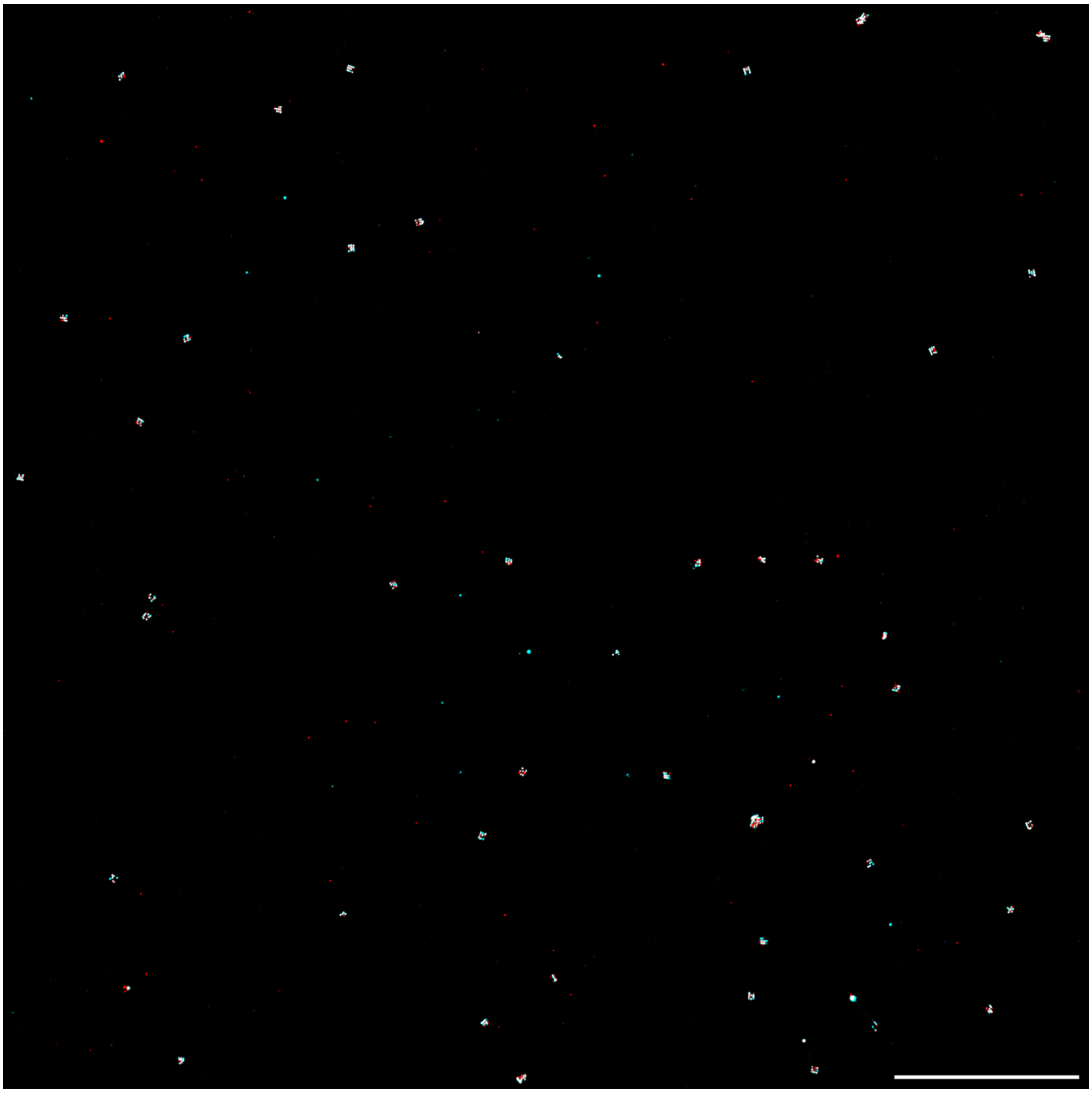

Figure S25 |Overview image of ALFA-tag nanoclusters templated at 20 nm distances. Antigen (ALFA-tag) position was imaged with Cy3B-labeled P3* imager strands (red) and the ALFA-tag nanobody was imaged with Cy3B-labeled P1* imager strands (cyan) using Exchange-PAINT. Scale bar: 2 µm

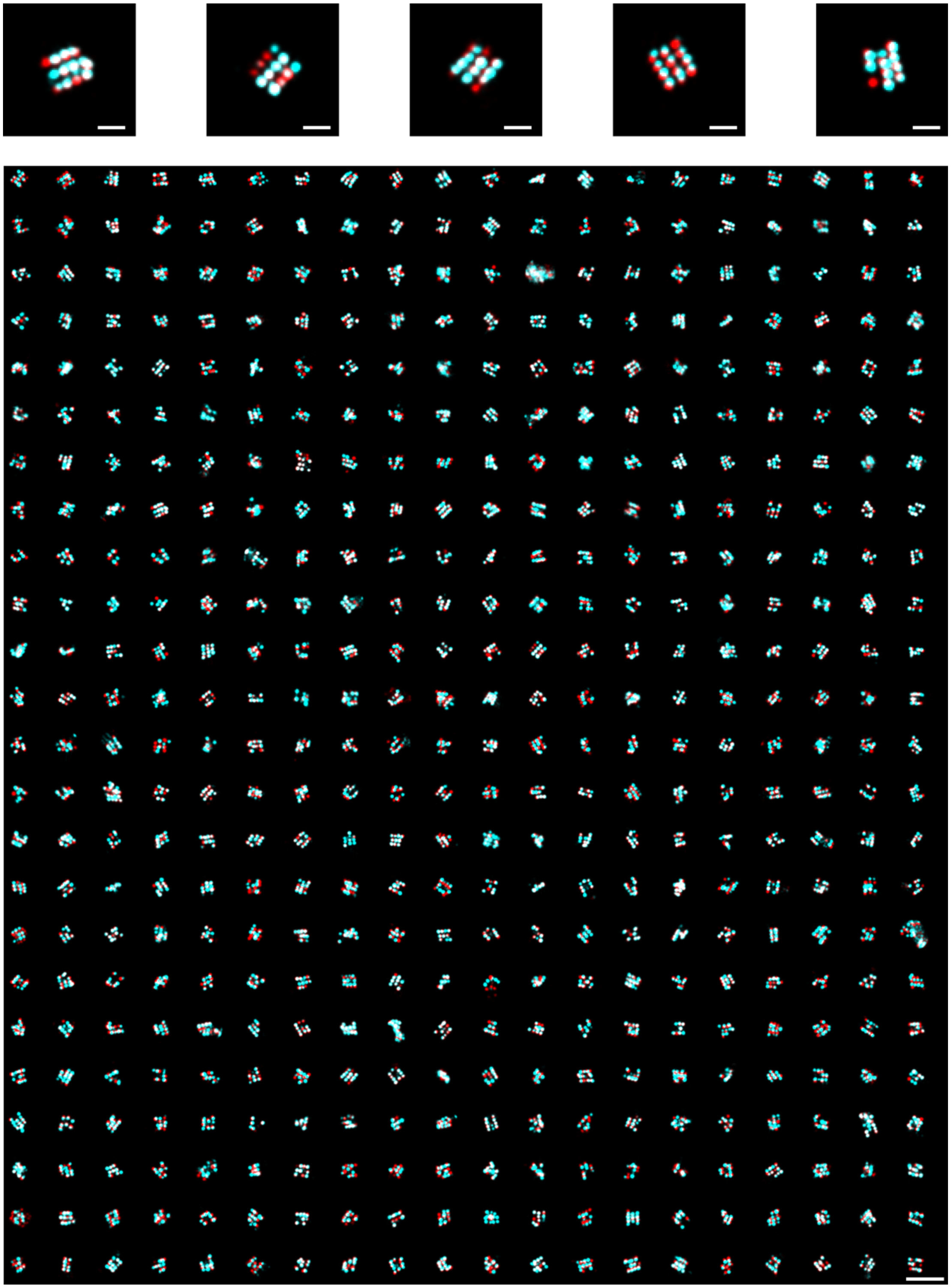

Figure S26 | Selected ALFA-tag nanoclusters templated at 20 nm distances for probe-based resolution estimate and labeling shadow determination. Five exemplary nanoclusters (top). Selected nanocluster for labeling shadow estimation (bottom). Antigen (ALFA-tag) position was imaged with Cy3B-labeled P3* imager strands (red) and ALFA-tag nanobody signal was imaged with Cy3B-labeled P1* imager strands (cyan) using Exchange-PAINT. Scale bars: 50 nm (top), 200 nm (bottom).

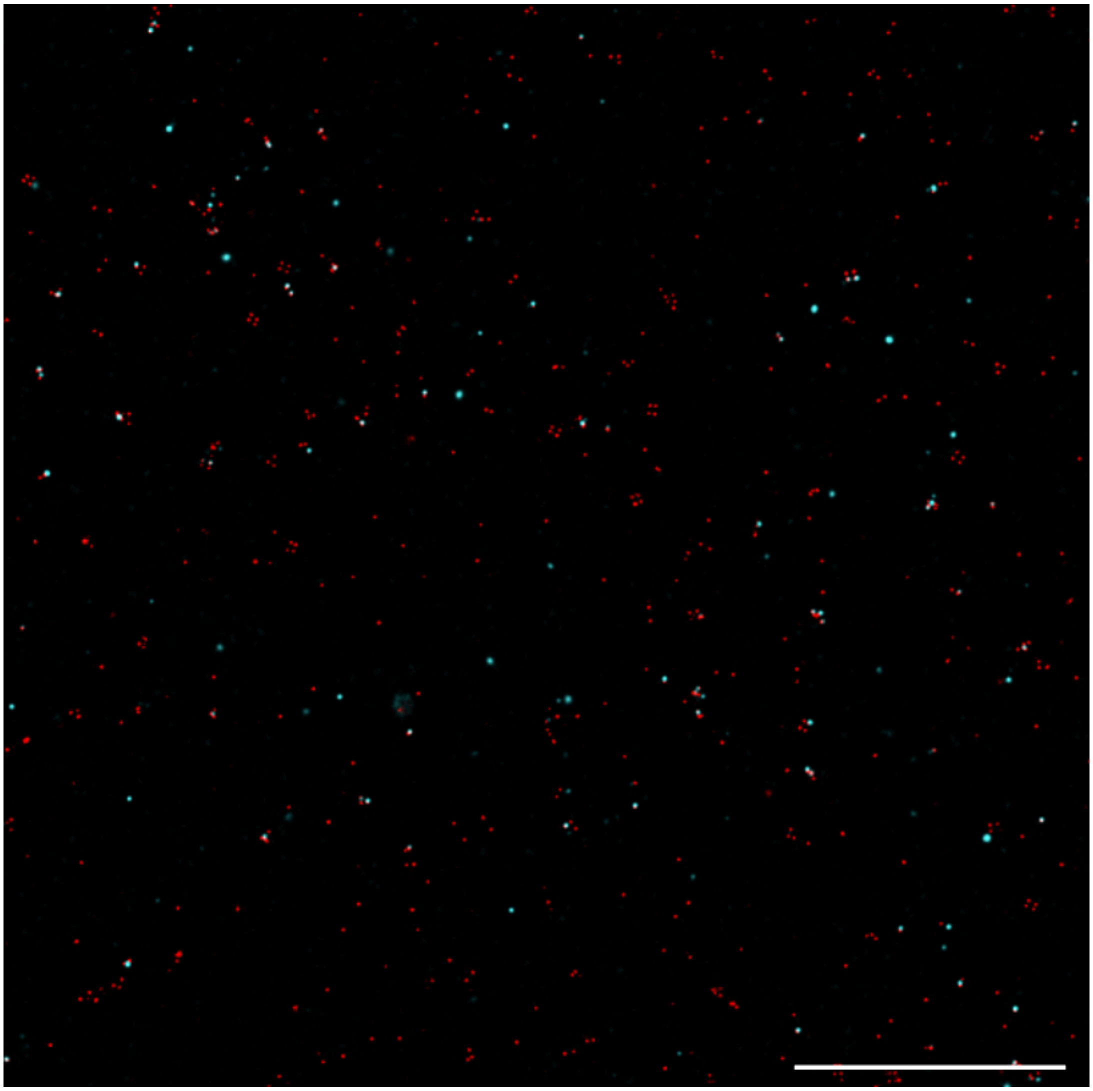

Figure S27 |Overview image of 1xCTD nanoclusters templated at 40x60 nm distances labeled with primary antibody and secondary nanobody. Antigen (1xCTD) position was imaged with Cy3B-labeled P3* imager strands (red) and the secondary nanobody that bound to CTD antibody was imaged with Cy3B-labeled P1* imager strands (cyan) using Exchange-PAINT. Scale bar: 2 µm

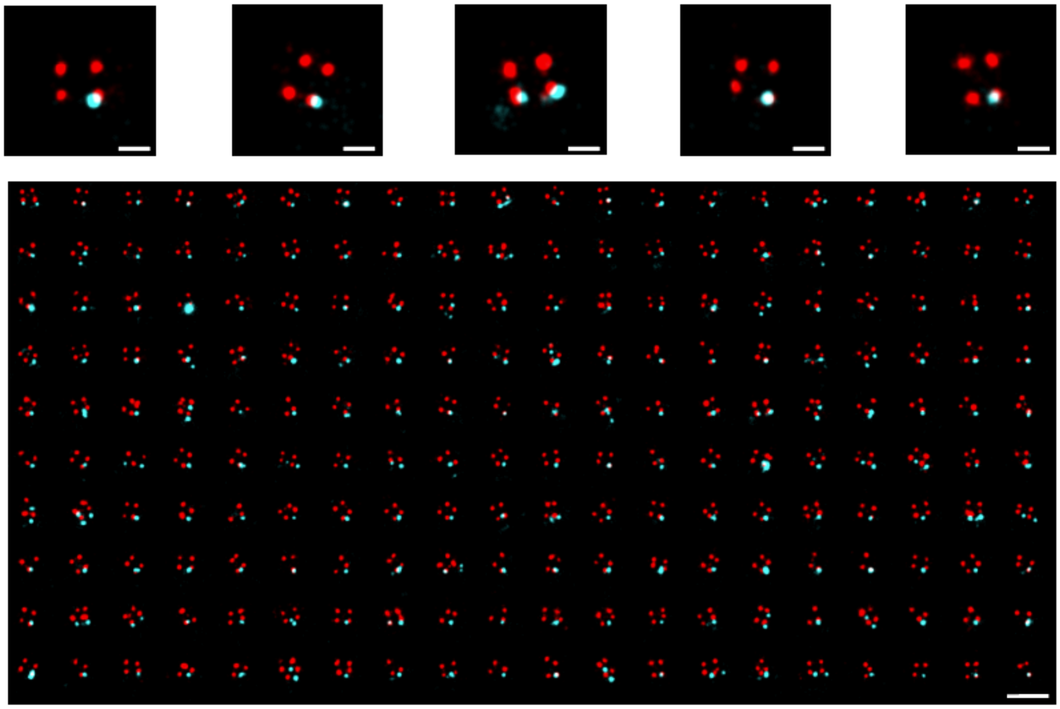

Figure S28 | Selected 1xCTD nanoclusters templated at 40x60 nm distances for probe-based resolution estimate and labeling shadow determination. Five exemplary nanoclusters (top). Selected nanocluster for labeling shadow estimation. Antigen (1xCTD) position was imaged with Cy3B-labeled P3* imager strands (red) and secondary nanobody that bound to primary CTD antibody signal was imaged with Cy3B-labeled P1* imager strands (cyan) using Exchange-PAINT. Scale bars: 50 nm (top), 200 nm (bottom)

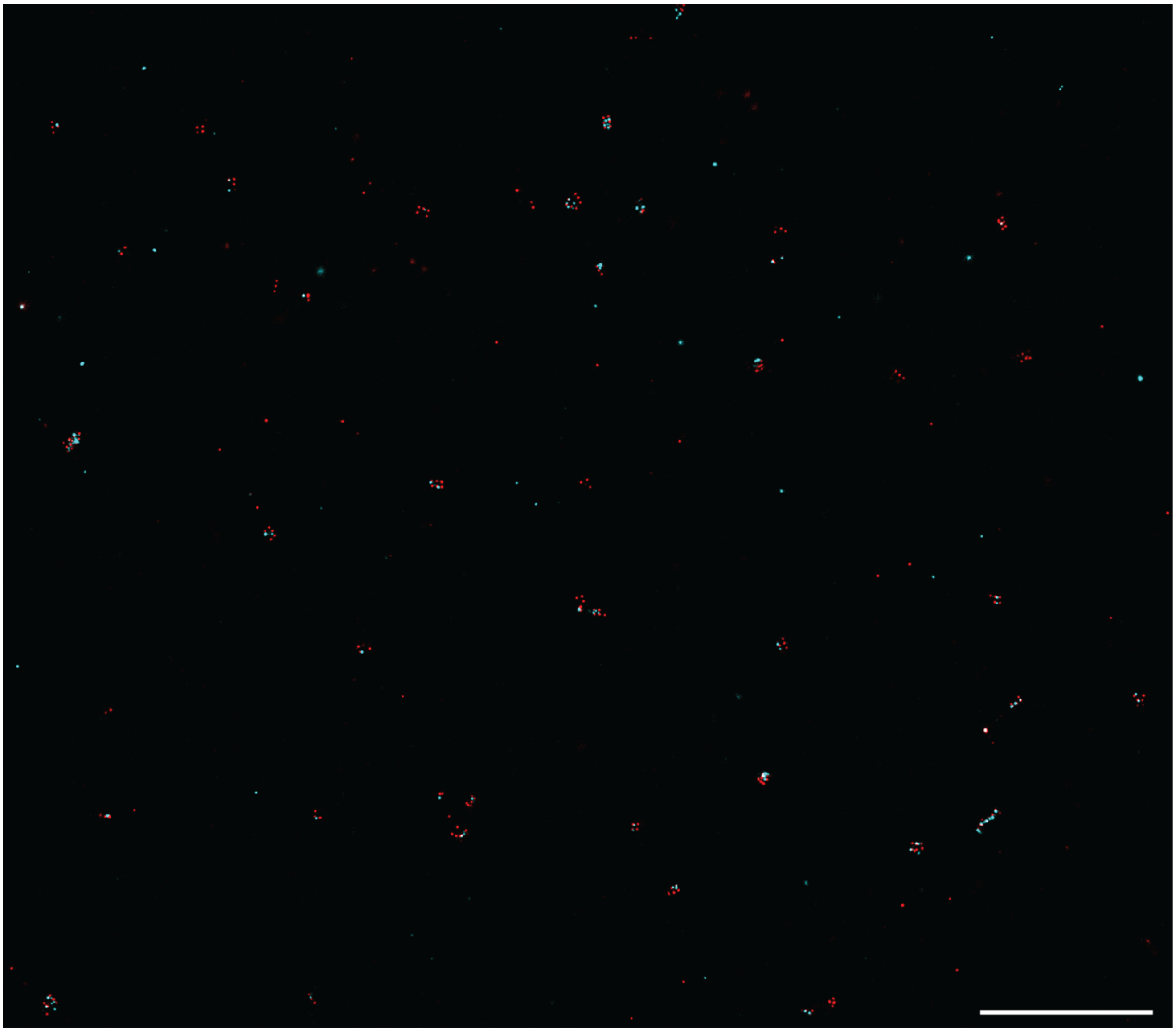

Figure S29 |Overview image of 1xCTD nanoclusters templated at 30 nm distances labeled with primary antibody and secondary nanobody. Antigen (1xCTD) position was imaged with Cy3B-labeled P3* imager strands (red) and the secondary nanobody that bound to CTD antibody was imaged with Cy3B-labeled P1* imager strands (cyan) using Exchange-PAINT. Scale bar: 1 µm

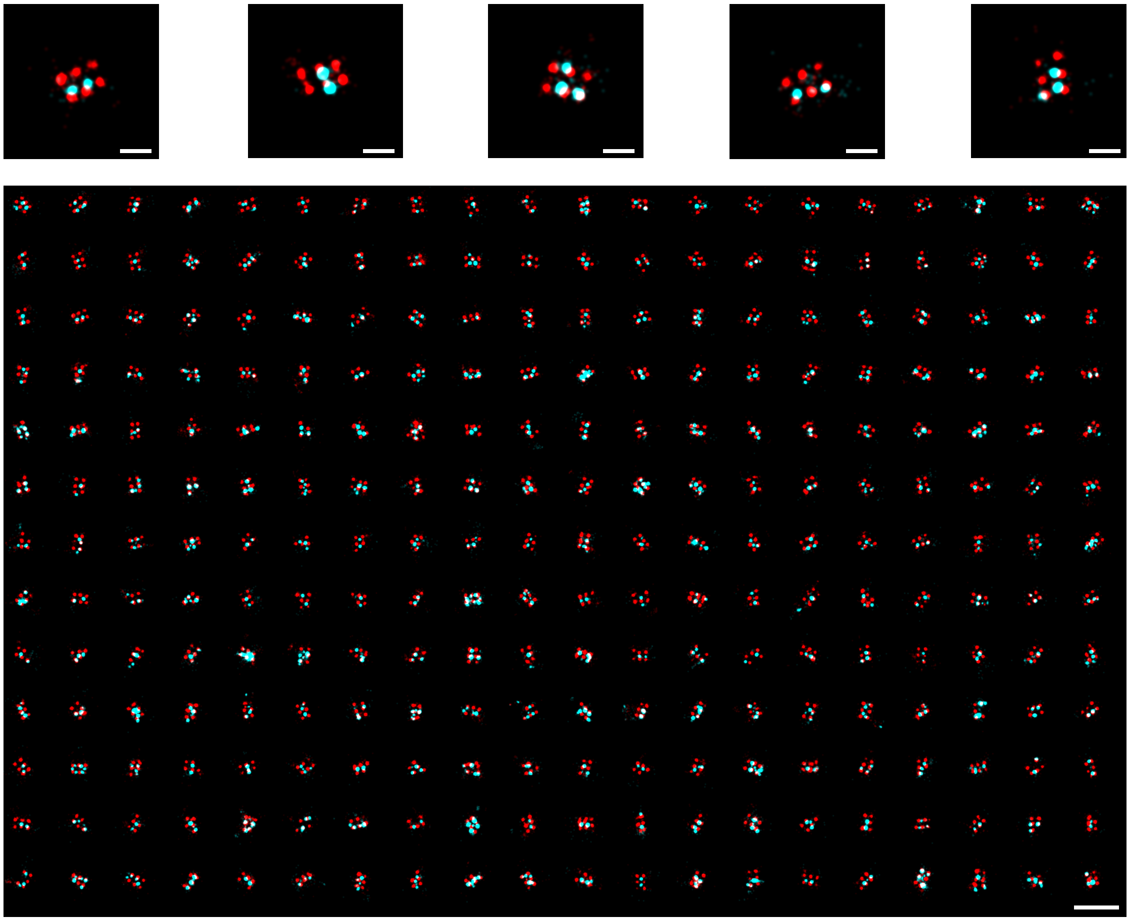

Figure S30 | Selected 1x CTD nanoclusters templated at 30 nm distances for probe-based resolution estimate and labeling shadow determination. Five exemplary nanoclusters (top). Selected nanocluster for labeling shadow estimation. Antigen (1xCTD) position was imaged with Cy3B-labeled P3* imager strands (red) and secondary nanobody that bound to primary CTD antibody signal was imaged with Cy3B-labeled P1* imager strands (cyan) using Exchange-PAINT. Scale bars: 50 nm (top), 200 nm (bottom)

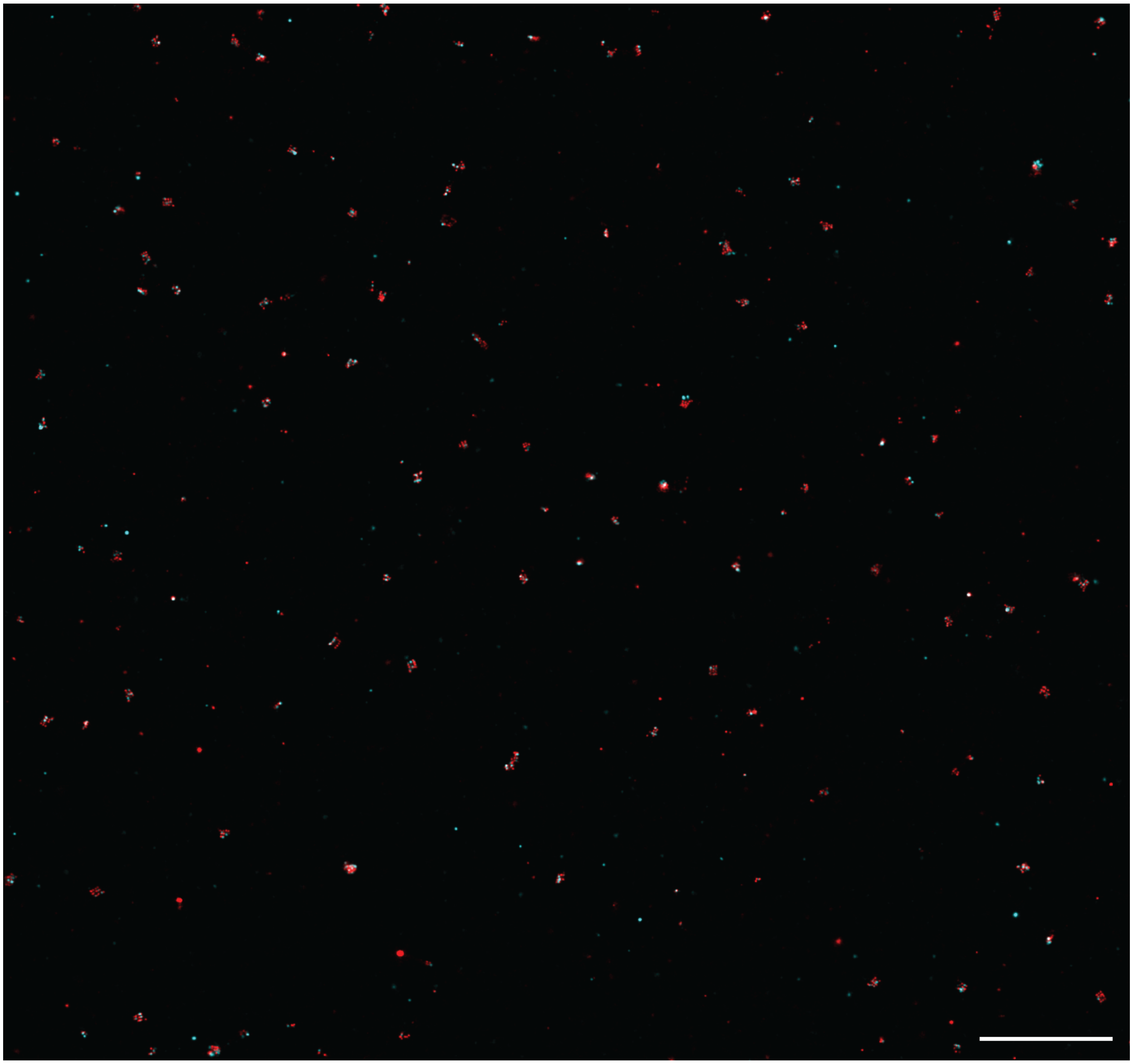

Figure S31 | Overview image of 1xCTD nanoclusters templated at 20 nm distances labeled with primary antibody and secondary nanobody. Antigen (1xCTD) position was imaged with Cy3B-labeled P3* imager strands (red) and the secondary nanobody that bound to CTD antibody was imaged with Cy3B-labeled P1* imager strands (cyan) using Exchange-PAINT. Scale bar: 1 µm

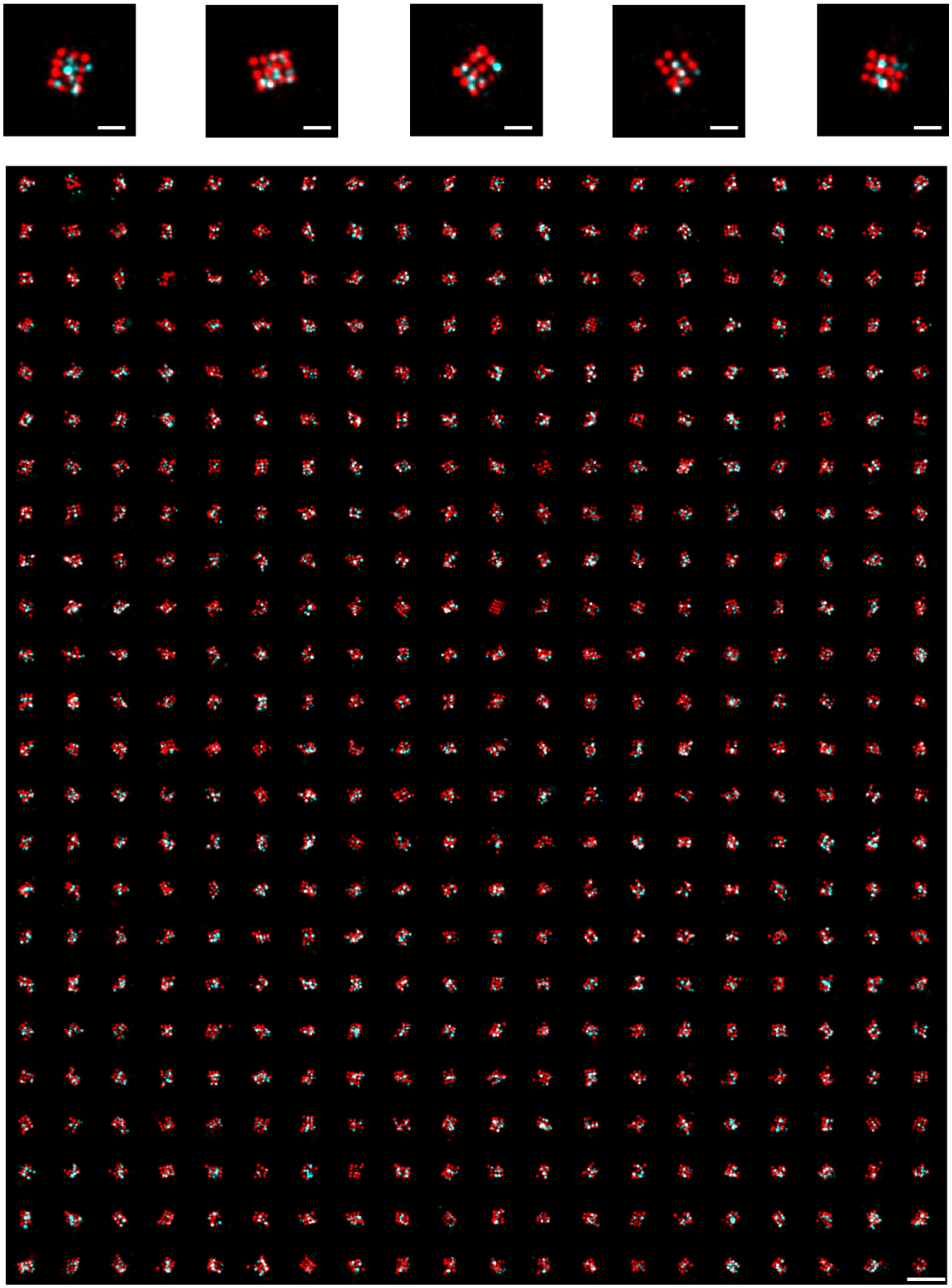

Figure S32 | Selected 1x CTD nanoclusters templated at 20 nm distances and labeled with primary antibody and secondary nanobody for probe-based resolution estimate and labeling shadow determination. Five exemplary nanoclusters (top). Selected nanocluster for labeling shadow estimation. Antigen (1xCTD) position was imaged with Cy3B-labeled P3* imager strands (red) and secondary nanobody that bound to CTD primary antibody was imaged with Cy3B-labeled P1* imager strands (cyan) using Exchange-PAINT. Scale bars: 50 nm (top), 200 nm (bottom)

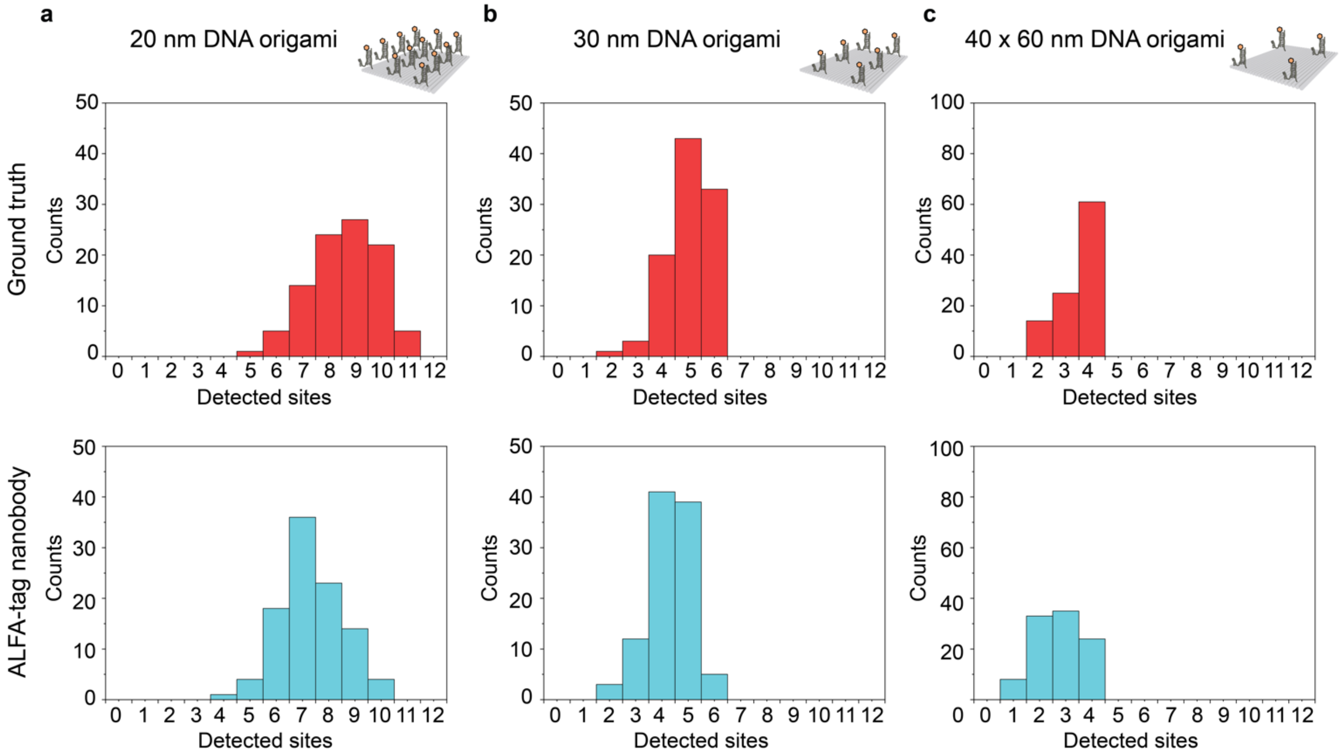

Figure S33 | Evaluation of Alfa-tag labeling efficiency on templated DNA origami antigen nanoclusters. Histogram comparison of detected underlying number of ground truth sites with the detected number of single ALFA-tag NB sites for 20 nm Origami **(a)**, 30 nm Origami **(b)** and 40 x 60 nm Origami **(c)**. (n=100 structures were counted for each pattern)

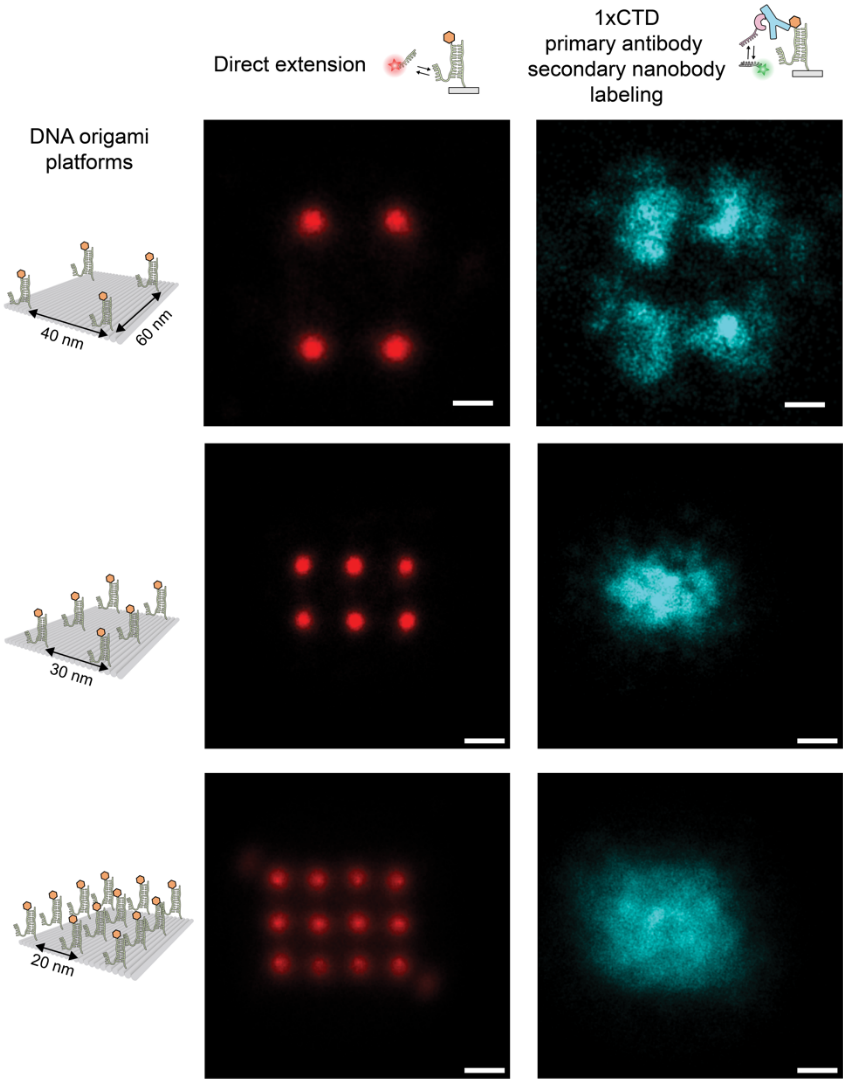

Figure S34 | DNA origami assessment of primary antibodies and secondary nanobodies. Schemes of DNA origami structures with positions of antigens (left). Schemes for DNA-PAINT imaging of antigen position (top middle), antibody positions bound to 1xCTD (top right). DNA origami sum images of antigen positions (red) and antibody positions (cyan). (n > 100 for each structure). Scale bars: 20 nm

Table S1 | DNA strands for DNA origami folding

Name	Sequence	20 nm structure	30 nm structure	40 nm structure
21[32]23[31]BLK	TTTTCACTCAAAGGGCGAAAAACCATCACC			
19[32]21[31]BLK	GTCGACTTCGGCCAACGCGCGGGGTTTTTC			
17[32]19[31]BLK	TGCATCTTTCCCAGTCACGACGGCCTGCAG			
15[32]17[31]BLK	TAATCAGCGGATTGACCGTAATCGTAACCG			
13[32]15[31]BLK	AACGCAAAATCGATGAACGGTACCGGTTGA			
11[32]13[31]BLK	AACAGTTTTGTACCAAAAACATTTTATTTC			
9[32]11[31]BLK	TTTACCCCAACATGTTTTAAATTTCCATAT			
7[32]9[31]BLK	TTTAGGACAAATGCTTTAAACAATCAGGTC			
5[32]7[31]BLK	CATCAAGTAAAACGAACTAACGAGTTGAGA			
3[32]5[31]BLK	AATACGTTTGAAAGAGGACAGACTGACCTT			
1[32]3[31]BLK	AGGCTCCAGAGGCTTTGAGGACACGGGTAA			
0[47]1[31]BLK	AGAAAGGAACAACTAAAGGAATTCAAAAAAA			
23[32]22[48]BLK	CAAATCAAGTTTTTTGGGGTCGAAACGTGGA			
22[47]20[48]BLK	CTCCAACGCAGTGAGACGGGCAACCAGCTGCA			
20[47]18[48]BLK	TTAATGAACTAGAGGATCCCCGGGGGGTAACG			
18[47]16[48]BLK	CCAGGGTTGCCAGTTTGAGGGGACCCGTGGGA			
16[47]14[48]BLK	ACAAACGGAAAAGCCCCAAAAACACTGGAGCA			
14[47]12[48]BLK	AACAAGAGGGATAAAAATTTTTAGCATAAAGC			
12[47]10[48]BLK	TAAATCGGGATTCCCAATTCTGCGATATAATG			
10[47]8[48]BLK	CTGTAGCTTGACTATTATAGTCAGTTCATTGA			
8[47]6[48]BLK	ATCCCCCTATACCACATTCAACTAGAAAAATC			
6[47]4[48]BLK	TACGTTAAAGTAATCTTGACAAGAACCGAACT			
4[47]2[48]BLK	GACCAACTAATGCCACTACGAAGGGGGTAGCA			
2[47]0[48]BLK	ACGGCTACAAAAGGAGCCTTTAATGTGAGAAT			
21[56]23[63]BLK	AGCTGATTGCCCTTCAGAGTCCACTATTAAAGGGTGCCGT			
15[64]18[64]BLK	GTATAAGCCAACCCGTCGGATTCTGACGACAGTATCGGCCGCAAGGCG			
13[64]15[63]BLK	TATATTTTGTCATTGCCTGAGAGTGGAAGATT			
11[64]13[63]BLK	GATTTAGTCAATAAAGCCTCAGAGAACCCTCA			
9[64]11[63]BLK	CGGATTGCAGAGCTTAATTGCTGAAACGAGTA			
7[56]9[63]BLK	ATGCAGATACATAACGGGAATCGTCATAAATAAAGCAAAG			
1[64]4[64]BLK	TTTATCAGGACAGCATCGGAACGACACCAACCTAAAACGAGGTCAATC			
0[79]1[63]BLK	ACAACTTTCAACAGTTTCAGCGGATGTATCGG			
23[64]22[80]BLK	AAAGCACTAAATCGGAACCCTAATCCAGTT			

22[79]20[80]BLK	TGGAACAACCGCCTGGCCCTGAGGCCCGCT			
20[79]18[80]BLK	TTCCAGTCGTAATCATGGTCATAAAAGGGG			
18[79]16[80]BLK	GATGTGCTTCAGGAAGATCGCACAATGTGA			
16[79]14[80]BLK	GCGAGTAAAAATATTTAAATTGTTACAAAG			
14[79]12[80]BLK	GCTATCAGAAATGCAATGCCTGAATTAGCA			
12[79]10[80]BLK	AAATTAAGTTGACCATTAGATACTTTTGCG			
10[79]8[80]BLK	GATGGCTTATCAAAAAGATTAAGAGCGTCC			
8[79]6[80]BLK	AATACTGCCCAAAAGGAATTACGTGGCTCA			
6[79]4[80]BLK	TTATACCACCAAATCAACGTAACGAACGAG			
4[79]2[80]BLK	GCGCAGACAAGAGGCAAAAGAATCCCTCAG			
2[79]0[80]BLK	CAGCGAAACTTGCTTTCGAGGTGTTGCTAA			
21[96]23[95]BLK	AGCAAGCGTAGGGTTGAGTGTTGTAGGGAGCC			
19[96]21[95]BLK	CTGTGTGATTGCGTTGCGCTCACTAGAGTTGC			
17[96]19[95]BLK	GCTTTCCGATTACGCCAGCTGGCGGCTGTTTC			
15[96]17[95]BLK	ATATTTTGGCTTTCATCAACATTATCCAGCCA			
13[96]15[95]BLK	TAGGTAAACTATTTTTGAGAGATCAAACGTTA			
11[96]13[95]BLK	AATGGTCAACAGGCAAGGCAAAGAGTAATGTG			
9[96]11[95]BLK	CGAAAGACTTTGATAAGAGGTCATATTTCGCA			
7[96]9[95]BLK	TAAGAGCAAATGTTTAGACTGGATAGGAAGCC			
5[96]7[95]BLK	TCATTCAGATGCGATTTTAAGAACAGGCATAG			
3[96]5[95]BLK	ACACTCATCCATGTTACTTAGCCGAAAGCTGC			
1[96]3[95]BLK	AAACAGCTTTTTGCGGGATCGTCAACACTAAA			
0[111]1[95]BLK	TAAATGAATTTTCTGTATGGGATTAATTTCTT			
23[96]22[112]BLK	CCCGATTTAGAGCTTGACGGGGAAAAAGAATA			
22[111]20[112]BLK	GCCCGAGAGTCCACGCTGGTTTGCAGCTAACT			
20[111]18[112]BLK	CACATTAAAATTGTTATCCGCTCATGCGGGCC			
18[111]16[112]BLK	TCTTCGCTGCACCGCTTCTGGTGCGGCCTTCC			
16[111]14[112]BLK	TGTAGCCATTAAAATTCGCATTAAATGCCGGA			
14[111]12[112]BLK	GAGGGTAGGATTCAAAAGGGTGAGACATCCAA			
12[111]10[112]BLK	TAAATCATATAACCTGTTTAGCTAACCTTTAA			
10[111]8[112]BLK	TTGCTCCTTTCAAATATCGCGTTTGAGGGGGT			
8[111]6[112]BLK	AATAGTAAACACTATCATAACCCTCATTGTGA			
6[111]4[112]BLK	ATTACCTTTGAATAAGGCTTGCCCAAATCCGC			
4[111]2[112]BLK	GACCTGCTCTTTGACCCCCAGCGAGGGAGTTA			
2[111]0[112]BLK	AAGGCCGCTGATACCGATAGTTGCGACGTTAG			
21[120]23[127]BLK	CCCAGCAGGCGAAAAATCCCTTATAAATCAAGCCGGCG			
15[128]18[128]BLK	TAAATCAAAATAATTCGCGTCTCGGAAACCAGGCAAAGGGAAGG			
13[128]15[127]BLK	GAGACAGCTAGCTGATAAATTAATTTTTGT			
11[128]13[127]BLK	TTTGGGGATAGTAGTAGCATTAAAAGGCCG			

9[128]11[127]BLK	GCTTCAATCAGGATTAGAGAGTTATTTTCA			
7[120]9[127]BLK	CGTTTACCAGACGACAAAGAAGTTTTGCCATAATTCGA			
1[128]4[128]BLK	TGACAACTCGCTGAGGCTTGCATTATACCAAGCGCGATGATAAA			
0[143]1[127]BLK	TCTAAAGTTTTGTCGTCTTTCCAGCCGACAA			
21[160]22[144]BLK	TCAATATCGAACCTCAAATATCAATTCCGAAA			
19[160]20[144]BLK	GCAATTCACATATTCCTGATTATCAAAGTGTA			
17[160]18[144]BLK	AGAAAACAAAGAAGATGATGAAACAGGCTGCG			
15[160]16[144]BLK	ATCGCAAGTATGTAAATGCTGATGATAGGAAC			
13[160]14[144]BLK	GTAATAAGTTAGGCAGAGGCATTTATGATATT			
11[160]12[144]BLK	CCAATAGCTCATCGTAGGAATCATGGCATCAA			
9[160]10[144]BLK	AGAGAGAAAAAAATGAAAATAGCAAGCAAACT			
7[160]8[144]BLK	TTATTACGAAGAACTGGCATGATTGCGAGAGG			
5[160]6[144]BLK	GCAAGGCCTCACCAGTAGCACCATGGGCTTGA			
3[160]4[144]BLK	TTGACAGGCCACCACCAGAGCCGCGATTTGTA			
1[160]2[144]BLK	TTAGGATTGGCTGAGACTCCTCAATAACCGAT			
0[175]0[144]BLK	TCCACAGACAGCCCTCATAGTTAGCGTAACGA			
23[128]23[159]BLK	AACGTGGCGAGAAAGGAAGGGAAACCAGTAA			
22[143]21[159]BLK	TCGGCAAATCCTGTTTGATGGTGGACCCTCAA			
20[143]19[159]BLK	AAGCCTGGTACGAGCCGGAAGCATAGATGATG			
18[143]17[159]BLK	CAACTGTTGCGCCATTCGCCATTCAAACATCA			
16[143]15[159]BLK	GCCATCAAGCTCATTTTTTAACCACAAATCCA			
14[143]13[159]BLK	CAACCGTTTCAAATCACCATCAATTCGAGCCA			
12[143]11[159]BLK	TTCTACTACGCGAGCTGAAAAGGTTACCGCGC			
10[143]9[159]BLK	CCAACAGGAGCGAACCAGACCGGAGCCTTTAC			
8[143]7[159]BLK	CTTTTGCAGATAAAAACCAAAATAAAGACTCC			
6[143]5[159]BLK	GATGGTTTGAACGAGTAGTAAATTTACCATTA			
4[143]3[159]BLK	TCATCGCCAACAAAGTACAACGGACGCCAGCA			
2[143]1[159]BLK	ATATTCGGAACCATCGCCCACGCAGAGAAGGA			
23[160]22[176]BLK	TAAAAGGGACATTCTGGCCAACAAAGCATC			
22[175]20[176]BLK	ACCTTGCTTGGTCAGTTGGCAAAGAGCGGA			
20[175]18[176]BLK	ATTATCATTCAATATAATCCTGACAATTAC			
18[175]16[176]BLK	CTGAGCAAAAATTAATTACATTTTGGGTTA			
16[175]14[176]BLK	TATAACTAACAAAGAACGCGAGAACGCCAA			
14[175]12[176]BLK	CATGTAATAGAATATAAAGTACCAAGCCGT			
12[175]10[176]BLK	TTTTATTTAAGCAAATCAGATATTTTTTGT			
10[175]8[176]BLK	TTAACGTCTAACATAAAAACAGGTAACGGA			
8[175]6[176]BLK	ATACCCAACAGTATGTTAGCAAATTAGAGC			
6[175]4[176]BLK	CAGCAAAAGGAAACGTCACCAATGAGCCGC			
4[175]2[176]BLK	CACCAGAAAGGTTGAGGCAGGTCATGAAAG			

2[175]0[176]BLK	TATTAAGAAGCGGGGTTTTGCTCGTAGCAT			
21[184]23[191]BLK	TCAACAGTTGAAAGGAGCAAATGAAAAATCTAGAGATAGA			
15[192]18[192]BLK	TCAAATATAACCTCCGGCTTAGGTAACAATTTCATTTGAAGGCGAATT			
13[192]15[191]BLK	GTAAAGTAATCGCCATATTTAACAAAACTTTT			
11[192]13[191]BLK	TATCCGGTCTCATCGAGAACAAGCGACAAAAG			
9[192]11[191]BLK	TTAGACGGCCAAATAAGAAACGATAGAAGGCT			
7[184]9[191]BLK	CGTAGAAAATACATACCGAGGAAACGCAATAAGAAGCGCA			
1[192]4[192]BLK	GCGGATAACCTATTATTCTGAAACAGACGATTGGCCTTGAAGAGCCAC			
0[207]1[191]BLK	TCACCAGTACAAACTACAACGCCTAGTACCAG			
23[192]22[208]BLK	ACCCTTCTGACCTGAAAGCGTAAGACGCTGAG			
22[207]20[208]BLK	AGCCAGCAATTGAGGAAGGTTATCATCATTTT			
20[207]18[208]BLK	GCGGAACATCTGAATAATGGAAGGTACAAAAT			
18[207]16[208]BLK	CGCGCAGATTACCTTTTTTAATGGGAGAGACT			
16[207]14[208]BLK	ACCTTTTTATTTTAGTTAATTTCATAGGGCTT			
14[207]12[208]BLK	AATTGAGAATTCTGTCCAGACGACTAAACCAA			
12[207]10[208]BLK	GTACCGCAATTCTAAGAACGCGAGTATTATTT			
10[207]8[208]BLK	ATCCCAATGAGAATTAACTGAACAGTTACCAG			
8[207]6[208]BLK	AAGGAAACATAAAGGTGGCAACATTATCACCG			
6[207]4[208]BLK	TCACCGACGCACCGTAATCAGTAGCAGAACCG			
4[207]2[208]BLK	CCACCCTCTATTCACAAACAAATACCTGCCTA			
2[207]0[208]BLK	TTTCGGAAGTGCCGTCGAGAGGGTGAGTTTCG			
21[224]23[223]BLK	CTTTAGGGCCTGCAACAGTGCCAATACGTG			
19[224]21[223]BLK	CTACCATAGTTTGAGTAACATTTAAAATAT			
17[224]19[223]BLK	CATAAATCTTTGAATACCAAGTGTTAGAAC			
15[224]17[223]BLK	CCTAAATCAAAATCATAGGTCTAAACAGTA			
13[224]15[223]BLK	ACAACATGCCAACGCTCAACAGTCTTCTGA			
11[224]13[223]BLK	GCGAACCTCCAAGAACGGGTATGACAATAA			
9[224]11[223]BLK	AAAGTCACAAAATAAACAGCCAGCGTTTTA			
7[224]9[223]BLK	AACGCAAAGATAGCCGAACAAACCCTGAAC			
5[224]7[223]BLK	TCAAGTTTCATTAAAGGTGAATATAAAAGA			
3[224]5[223]BLK	TTAAAGCCAGAGCCGCCACCCTCGACAGAA			
1[224]3[223]BLK	GTATAGCAAACAGTTAATGCCCAATCCTCA			
0[239]1[223]BLK	AGGAACCCATGTACCGTAACACTTGATATAA			
23[224]22[240]BLK	GCACAGACAATATTTTTGAATGGGGTCAGTA			
22[239]20[240]BLK	TTAACACCAGCACTAACAACTAATCGTTATTA			
20[239]18[240]BLK	ATTTTAAAATCAAAATTATTTGCACGGATTCG			
18[239]16[240]BLK	CCTGATTGCAATATATGTGAGTGATCAATAGT			

16[239]14[240]BLK	GAATTTATTTAATGGTTTGAAATATTCTTACC			
14[239]12[240]BLK	AGTATAAAGTTCAGCTAATGCAGATGTCTTTC			
12[239]10[240]BLK	CTTATCATTCCCGACTTGCGGGAGCCTAATTT			
10[239]8[240]BLK	GCCAGTTAGAGGGTAATTGAGCGCTTTAAGAA			
8[239]6[240]BLK	AAGTAAGCAGACACCACGGAATAATATTGACG			
6[239]4[240]BLK	GAAATTATTGCCTTTAGCGTCAGACCGGAACC			
4[239]2[240]BLK	GCCTCCCTCAGAATGGAAAGCGCAGTAACAGT			
2[239]0[240]BLK	GCCCGTATCCGGAATAGGTGTATCAGCCCAAT			
21[248]23[255]BLK	AGATTAGAGCCGTCAAAAAACAGAGGTGAGGCCTATTAGT			
15[256]18[256]BLK	GTGATAAAAAGACGCTGAGAAGAGATAACCTTGCTTCTGTTCGGGAGA			
13[256]15[255]BLK	GTTTATCAATATGCGTTATACAAACCGACCGT			
11[256]13[255]BLK	GCCTTAAACCAATCAATAATCGGCACGCGCCT			
9[256]11[255]BLK	GAGAGATAGAGCGTCTTTCCAGAGGTTTTGAA			
7[248]9[255]BLK	GTTTATTTTGTCACAATCTTACCGAAGCCCTTTAATATCA			
1[256]4[256]BLK	CAGGAGGTGGGGTCAGTGCCTTGAGTCTCTGAATTTACCGGGAACCAG			
0[271]1[255]BLK	CCACCCTCATTTTCAGGGATAGCAACCGTACT			
23[256]22[272]BLK	CTTTAATGCGCGAACTGATAGCCCCACCAG			
22[271]20[272]BLK	CAGAAGATTAGATAATACATTTGTCGACAA			
20[271]18[272]BLK	CTCGTATTAGAAATTGCGTAGATACAGTAC			
18[271]16[272]BLK	CTTTTACAAAATCGTCGCTATTAGCGATAG			
16[271]14[272]BLK	CTTAGATTTAAGGCGTTAAATAAAGCCTGT			
14[271]12[272]BLK	TTAGTATCACAATAGATAAGTCCACGAGCA			
12[271]10[272]BLK	TGTAGAAATCAAGATTAGTTGCTCTTACCA			
10[271]8[272]BLK	ACGCTAACACCCACAAGAATTGAAAATAGC			
8[271]6[272]BLK	AATAGCTATCAATAGAAAATTCAACATTCA			
6[271]4[272]BLK	ACCGATTGTCGGCATTTTCGGTCATAATCA			
4[271]2[272]BLK	AAATCACCTTCCAGTAAGCGTCAGTAATAA			
2[271]0[272]BLK	GTTTTAACTTAGTACCGCCACCCAGAGCCA			

Table S2 | Additional DNA reagents for imaging and peptide attachment

Name	Sequence	5'-Mod	3'-Mod
Extension on DNA origami for peptide attachment	5'-staple-TTGTGATGTAGGTGGTAGAGGAA-3'	Staple	
Peptide attachment strand (with P3 handle)	5'-DBCO-TTCCTCTACCACCTACATCACTTTCTTCATTA-3'	DBCO	
Extension on DNA origami for qPAINT calibration (P1)	5'-staple-TT ATACATCTA-3'	Staple	
Control Strand (P1 & P3 handle)	ATACATCTATTTTC CTC TAC CAC CTA CAT CAC TTT CTT CAT TA		
P1 – docking strand	5'-Azide-TT ATACATCTA-3'	Azide	
P1* imager strand	5'-CTAGATGTAT-Cy3B-3'		Cy3B
P3* imager strand	5'-GTAATGAAGA-Cy3B-3'		Cy3B

Table S3 | Peptide reagents

Name	Sequence	Modification
1xALFA-Tag	Azide-Lysine- SRLEEELRRRLTE	Azide
1xCTD	Azide-Lysine - YSPTS(p)PS	Azide
5xCTD	Azide-Lysine - YSPTS(p)PSYSPTS(p)PSYSPTS(p)PSYSPTS(p)PSYSPTS(p)PS	Azide

Table S4 | Imaging conditions

Dataset	Parameters	Power	NeNa
Cell imaging<br>Supp. Figure 1	30,000 frames, P1, 100 pM, 150 ms, Cy3B	220 W/cm^2	5.4 nm
Digoxigenin SM Experiment,<br>Figure 1<br>Supp. Figure: 2, 3	Binder: 15,000 Frames, P1, 500pM, 100ms, Cy3b<br>TruePos: 15,000 Frames, P3, 2.5nM, 100ms, Atto647N	48 W/cm^2<br>72 W/ cm^2	4.9 nm<br>10.4 nm
1xCTD SM Experiment<br>Figure 1<br>Supp. Figures: 4, 5	Binder: 15,000 Frames, P1, 2.5nM, 100ms, Cy3b<br>TruePos: 15,000 Frames, P3, 2.5nM, 100ms, Atto647N	120 W/cm^2<br>168 W/cm^2	4.2 nm<br>8.7 nm
5xCTD SM Experiment<br>Figure 1<br>Supp. Figures 6, 7	Binder: 15,000 Frames, P1, 100pM, 100ms, Cy3b<br>TruePos: 15,000 Frames, P3, 2.5nM, 100ms, Atto647N	144 W/cm^2<br>144 W/cm^2	4.8 nm<br>9.7 nm
1xCTD SM Experiment<br>Secondary nanobody<br>Supp. Figure 8	Binder: 15,000 Frames, P1, 2.5nM, 100ms, Cy3b<br>TruePos: 15,000 Frames, P3, 2.5nM, 100ms, Atto647N	200 W/cm^2<br>169 W/cm^2	4.6 nm<br>8.8 nm
1xALFA SM<br>Experiment<br>Figure 1<br>Supp. Figures 9, 10	Binder: 20,000 Frames, P1, 4nM, 100ms, Cy3b<br>TruePos: 18,177 Frames, P3, 4nM, 100ms, Atto647N	384 W/cm^2<br>384 W/cm^2	4.3 nm<br>6.4 nm
Direct Extension SM<br>Experiment<br>Figure 1<br>Supp. Figures 11, 12	Direct Ext.: 15,000 Frames, P1, 2.5nM, 100ms, Cy3b<br>TruePos: 15,000 Frames, P3, 1nM, 100ms, Atto647N	120 W/cm^2<br>110 W/cm^2	7.5 nm<br>10.3 nm
ALFAtag qPAINT<br>Experiment<br>Figure 1<br>Supp. Figure 13	Binder: 30,000 Frames, P1, 2.5nM, 200ms, Cy3b<br>TruePos: 15,000 Frames, P3, 5nM, 100ms, Cy3b	14 W/cm^2<br>120 W/cm^2	10.9 nm<br>7.1 nm
1xCTD qPAINT (1:10)<br>Experiment<br>Figure 1	Binder: 20,000 Frames, P1, 500pM, 100ms, Cy3b<br>TruePos: 15,000 Frames, P3, 5nM, 100ms, Cy3b	24 W/cm^2<br>143 W/cm^2	10.4 nm<br>7.8 nm
1xCTD qPAINT<br>(1:5) Experiment<br>Figure 1	Binder: 15,000 Frames, P1, 1nM, 200ms, Cy3b<br>TruePos: 15,000 Frames, P3, 5nM, 100ms, Cy3b	58 W/cm^2<br>72 W/cm^2	4.5 nm<br>7.8 nm
1xCTD qPAINT<br>(1:2) Experiment<br>Figure 1	Binder: 15,000 Frames, P1, 1nM, 200ms, Cy3b<br>TruePos: 15,000 Frames, P3, 5nM, 100ms, Cy3b	288 W/cm^2<br>288 W/cm^2	4.1 nm<br>4.9 nm
20nm ALFAtag<br>nanoclusters<br>Figure 2<br>Supp. Figures: 25, 26	Binder: 20,000 Frames, P1, 2.5 nM, 100ms, Cy3b<br>TruePos: 30,000 Frames, P3, 5 nM, 100ms, Cy3b	288 W/cm^2<br>288 W/cm^2	4.4 nm<br>4.7 nm
20nm 1xCTD nanoclusters<br>Figure 2<br>Supp. Figures: 19, 20	Binder: 20,000 Frames, P1, 2.5 nM, 100ms, Cy3b<br>TruePos: 20,002 Frames, P3, 5 nM, 100ms, Cy3b	288 W/cm^2<br>288 W/cm^2	4.6 nm<br>5.0 nm
20nm 1xCTD secondary<br>nanobody<br>Nanoclusters<br>Supp. Figures: 31, 32	Binder: 20,000 Frames, P1, 2.5 nM, 100ms, Cy3b<br>TruePos: 20,000 Frames, P3, 5nM, 100ms, Cy3b	324 W/cm^2<br>324 W/cm^2	4.5 nm<br>4.8 nm
30nm ALFAtag<br>Nanoclusters<br>Figure 2<br>Supp. Figures: 23, 24	Binder: 15,000 Frames, P1, 5nM, 100ms, Cy3b<br>TruePos: 15,000 Frames, P3, 5nM, 100ms, Cy3b	480 W/cm^2<br>480 W/cm^2	4.5 nm<br>5.1 nm
30nm 1xCTD nanoclusters<br>Figure 2<br>Supp. Figures: 17, 18	Binder: 15,000 Frames, P1, 1 nM, 100ms, Cy3b<br>TruePos: 15,000 Frames, P3, 5 nM, 100ms, Cy3b	480 W/cm^2<br>480 W/cm^2	3.6 nm<br>5.6 nm

30nm 1xCTD secondary nanobody Nanoclusters<br>Supp. Figures: 29, 30	Binder: 15,000 Frames, P1, 5 nM, 100ms, Cy3b<br>TruePos: 15,000 Frames, P3, 5 nM, 100ms, Cy3b	494 W/cm^2<br>494 W/cm^2	3.8 nm<br>3.7 nm
40x60nm 1xALFAtag nanoclusters<br>Figure 2<br>Supp. Figures: 21, 22	Binder: 15,000 Frames, P1, 2 nM, 100ms, Cy3b<br>TruePos: 15,000 Frames, P3, 5nM, 100ms, Cy3b	576 W/cm^2<br>576 W/cm^2	5.1 nm<br>5.0 nm
40x60nm 1xCTD nanoclusters<br>Figure 2<br>Supp. Figures: 15, 16	Binder: 15,000 Frames, P1, 500 pM, 100ms, Cy3b<br>TruePos: 15,000 Frames, P3, 2 nM, 100ms, Cy3b	576 W/cm^2<br>576 W/cm^2	3.7 nm<br>5.1 nm
40x60nm 1xCTD secondary nanobody Nanoclusters<br>Supp. Figures: 27, 28	Binder: 15,000 Frames, P1, 2 nM, 100ms, Cy3b<br>TruePos: 15,000 Frames, P3, 5 nM, 100ms, Cy3b	325 W/cm^2<br>325 W/cm^2	4.9 nm<br>4.7 nm
